# Reduced free asparagine in wheat grain resulting from a natural deletion of *TaASN-B2*: investigating and exploiting diversity in the asparagine synthetase gene family to improve wheat quality

**DOI:** 10.1186/s12870-021-03058-7

**Published:** 2021-06-29

**Authors:** Joseph Oddy, Rocío Alarcón-Reverte, Mark Wilkinson, Karl Ravet, Sarah Raffan, Andrea Minter, Andrew Mead, J. Stephen Elmore, Isabel Moreira de Almeida, Nicholas C. Cryer, Nigel G. Halford, Stephen Pearce

**Affiliations:** 1 Plant Sciences Department, Rothamsted Research, Harpenden, Hertfordshire, AL5 2JQ UK; 2 Department of Soil and Crop Sciences, Colorado State University, Fort Collins, CO 80523 USA; 3 Computational and Analytical Sciences Department, Rothamsted Research, Harpenden, Hertfordshire, AL5 2JQ UK; 4 Department of Food & Nutritional Sciences, University of Reading, Whiteknights, Reading, RG6 6DZ UK; 5 Mondelēz R&D International, Paris Saclay Tech Center, 6 Rue René Razel, 91400 Saclay, France; 6 Mondelēz UK R&D Ltd, Bournville Lane, Bournville, Birmingham, B30 2LU UK

**Keywords:** Wheat, *Triticum aestivum*, Asparagine metabolism, Acrylamide, Asparagine synthetase, Food safety, Wheat breeding

## Abstract

**Background:**

Understanding the determinants of free asparagine concentration in wheat grain is necessary to reduce levels of the processing contaminant acrylamide in baked and toasted wheat products. Although crop management strategies can help reduce asparagine concentrations, breeders have limited options to select for genetic variation underlying this trait. Asparagine synthetase enzymes catalyse a critical step in asparagine biosynthesis in plants and, in wheat, are encoded by five homeologous gene triads that exhibit distinct expression profiles. Within this family, *TaASN2* genes are highly expressed during grain development but *TaASN-B2* is absent in some varieties.

**Results:**

Natural genetic diversity in the asparagine synthetase gene family was assessed in different wheat varieties revealing instances of presence/absence variation and other polymorphisms, including some predicted to affect the function of the encoded protein. The presence and absence of *TaASN-B2* was determined across a range of UK and global common wheat varieties and related species, showing that the deletion encompassing this gene was already present in some wild emmer wheat genotypes. Expression profiling confirmed that *TaASN2* transcripts were only detectable in the grain, while *TaASN3.1* genes were highly expressed during the early stages of grain development. *TaASN-A2* was the most highly expressed *TaASN2* homeologue in most assayed wheat varieties. *TaASN-B2* and *TaASN-D2* were expressed at similar, lower levels in varieties possessing *TaASN-B2*. Expression of *TaASN-A2* and *TaASN-D2* did not increase to compensate for the absence of *TaASN-B2,* so total *TaASN2* expression was lower in varieties lacking *TaASN-B2*. Consequently, free asparagine concentrations in field-produced grain were, on average, lower in varieties lacking *TaASN-B2*, although the effect was lost when free asparagine accumulated to very high concentrations as a result of sulphur deficiency.

**Conclusions:**

Selecting wheat genotypes lacking the *TaASN-B2* gene may be a simple and rapid way for breeders to reduce free asparagine concentrations in commercial wheat grain.

**Supplementary Information:**

The online version contains supplementary material available at 10.1186/s12870-021-03058-7.

## Background

Asparagine in its free (soluble, non-protein) form is an important nitrogen transport and storage molecule in plants (see [1] for review). It also accumulates during abiotic and biotic stress and has potential roles in ammonia detoxification and reactive oxygen species/nitrous oxide production (see [2] for review). However, free asparagine is also the precursor for acrylamide (C_3_H_5_NO), a carcinogenic contaminant that forms during the frying, roasting, baking, toasting and high-temperature processing of grains, tubers, beans and storage roots (reviewed in [3]). Acrylamide is classified as an extremely hazardous substance in the United States (USA), a serious health hazard with acute toxicity in the European Union (EU), and a Group 2A carcinogen (probably carcinogenic to humans) by the International Agency for Research on Cancer [4].

The European Commission has led the way in developing a regulatory system for acrylamide levels in food (see [3] for a comprehensive review). The current EU regulation on acrylamide in food (Commission Regulation (EU) 2017/2158 [5]) states that acrylamide in food ‘potentially increases the risk of developing cancer for consumers in all age groups’. It also sets Benchmark Levels for acrylamide in different food types, and carries an explicit threat to set Maximum Levels (i.e. levels above which it would be illegal to sell a product) in the future.

In the USA, the federal government has not introduced equivalent regulations, although the Food and Drug Administration (FDA) has issued an acrylamide ‘action plan’ [6]. However, as long ago as 2005 the Attorney General of the State of California filed a lawsuit against five food companies and four restaurant chains for failing to label their products with a warning to alert consumers to the presence of acrylamide (reviewed in [3]). California has also seen private lawsuits brought against the coffee industry over the lack of warning notices. Regulators in other countries that have taken a position on dietary acrylamide include Health Canada, Food Standards Australia New Zealand (FSANZ) and authorities in Japan and Hong Kong (reviewed in [3]). Meanwhile, a recent study identified a unique mutational ‘signature’ associated with acrylamide and its metabolite, glycidamide, that was found at high frequency in multiple human tumour types [7]. This represents the strongest evidence yet of a link between dietary acrylamide intake and cancer in humans.

Reduced free asparagine concentrations in crop raw materials would greatly assist the food industry in complying with regulations on acrylamide in food products. In wheat, free asparagine concentrations in the grain are typically higher in plants grown in sulphur-deficient soils, or in plants infected by pathogens. Therefore, crop management strategies, including ensuring that wheat is supplied with sufficient sulphur during cultivation [8], and protected from pathogen infection [9, 10], are the most common strategies to reduce free asparagine concentrations. Nevertheless, wheat breeders are under pressure from food businesses to develop varieties with reduced concentrations of free asparagine in the grain. Although free asparagine concentrations do vary across genotypes and exhibit reasonably high heritability [11–13], the large effect of crop management and other environmental (E) factors, both per se and in combination with genetic factors (G × E), means that breeding for low asparagine concentrations will not be a simple task (reviewed in [3]). Through association mapping, several quantitative trait loci (QTL) for asparagine content have been identified, but no common QTL have been identified between studies [12, 13].

Asparagine is synthesised by the transfer of an amino group from glutamine to aspartate to make asparagine and glutamate in a reaction catalysed by asparagine synthetase. The cereal asparagine synthetase gene family comprises between two and five genes per diploid genome [14], with members of the Triticeae tribe all having five genes per genome, assigned to four groups: 1, 2, 3 (subdivided into 3.1 and 3.2) and 4. The one documented exception is that some hexaploid common wheat (*Triticum aestivum* L.; genomes AABBDD) and tetraploid emmer wheat (*T. turgidum*; genomes AABB) genotypes lack a group 2 gene in the B genome (*TaASN-B2*/*TdASN-B2*) [14, 15]. This gene is absent from the IWGSC RefSeq v1.1 genome assembly of the common wheat landrace Chinese Spring but is present in the cultivar Cadenza [14]. In tetraploid wheats, *ASN-B2* is absent in wild emmer wheat (*T. turgidum* ssp. *dicoccoides*) genotype Zavitan, but present in domesticated durum wheat cultivar Svevo (*T. turgidum* L. ssp. *durum* (Desf.) Husn.) [14]. Since wild emmer wheat is believed to be the B genome donor for both tetraploid durum and hexaploid common wheat [16], the most likely explanation for the presence of *ASN-B2* in some cultivars but not others is that the hybridisation event that produced hexaploid wheat occurred more than once, involving emmer wheats with and without *ASN-B2* [14]. This would be consistent with evidence of multiple hybridisations found from wider analyses of genome data [17].

The extent of the presence/absence of *ASN-B2* in different genotypes is particularly interesting because the *TaASN2* genes are the most highly expressed members of the asparagine synthetase family in the grain [18, 19], and their expression in the embryo is likely a key factor determining free asparagine concentrations in the grain as a whole [19]. Wheat plants carrying CRISPR/Cas9 induced edits in all six *TaASN2* alleles exhibit greatly reduced free asparagine concentration in their grains [20]. It follows that the natural *ASN-B2* deletion could represent a valuable genetic variant for wheat breeders to exploit in order to reduce free asparagine content in the grain. In the present study, therefore, natural genetic variation in the asparagine synthetase gene family in wheat was characterised. The presence/absence of *ASN-B2* was screened in a panel of UK and global common wheat varieties, as well as wheat progenitor genomes and wild species. The deletion of *TaASN-B2* is associated with an overall reduction in *TaASN2* transcript levels and grain asparagine concentrations and may be a useful allele for wheat breeding programmes to develop varieties with lower concentrations of free asparagine.

## Results

### Natural diversity in the asparagine synthetase gene family in wheat

Full-length coding sequences of *ASN* genes from the wheat landrace Chinese Spring were used as queries in BLASTn searches against the genome assemblies of 14 common wheat varieties and spelt wheat (*T. aestivum* ssp. *spelta*) to characterise natural allelic variation in the wheat asparagine synthetase gene family. The results are shown in Table 1, ordered by gene name [14, 15] and the corresponding annotated gene model ID from the Chinese Spring RefSeq v1.1 genome assembly [21]. For each orthologous gene, Sorting Intolerant From Tolerant (SIFT) analysis was performed on the translated protein to predict whether the variation in amino acid sequences was likely to disrupt protein function (highlighted in yellow in Table 1) or to be tolerated (highlighted in green). Full details of specific amino acid changes for all wheat varieties are provided in Additional file 1, Table S1.

**Table 1:**
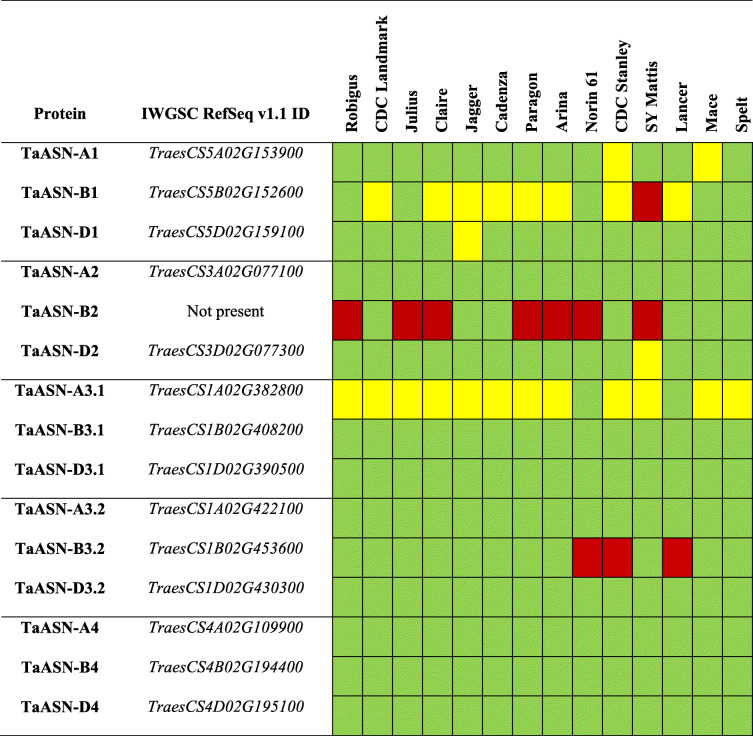
Natural variation in ASN proteins in 14 wheat varieties. Each gene is annotated by name [14, 15] and genome (hence *TaASN-A1*, for example, is the A genome *TaASN1* gene), and the gene ID from the Chinese Spring RefSeq v1.1 gene models [18] (note that *TaASN-B2* is absent in Chinese Spring). Green shading indicates no predicted impact of variety-specific mutations; yellow shading indicates one or more mutations that are predicted to disrupt protein function; red shading indicates that the gene is not present in that genome.

There were deletions, polymorphisms and presence/absence variation in several wheat *ASN* genes. For example, *TaASN-B1* was deleted in SY Mattis, while eight other varieties carried an allelic variant with a 16 bp deletion in exon seven, introducing a frame shift and bringing a premature stop codon into frame. The presence of this deletion means that the gene is predicted to encode a 375 amino acid protein with a C-terminal truncation of 209 amino acids, including part of the asparagine synthetase domain, indicating that this protein is likely to be non-functional (Additional file 1, Fig. S1). *TaASN-B3.2* was deleted in Norin 61, CDC Stanley and Lancer, whereas *TaASN-A3.2* and *TaASN-D3.2* were present in all analysed varieties, and showed no polymorphisms predicted to impact protein function (Table 1). In contrast, 12 wheat varieties carried *TaASN-A3.1* alleles with polymorphisms predicted to disrupt protein function (Table 1). Some varieties carry a combination of alleles predicted to disrupt the function of multiple asparagine synthetase proteins. For example, CDC Stanley carries alleles predicted to affect the function of the enzymes encoded by *TaASN-A1*, *TaASN-B1* and *TaASN-A3.1*, in addition to a deletion of *TaASN-B3.2*, while SY Mattis carries deletions of *TaASN-B1* and *TaASN-B2*, and disruptive alleles of *TaASN-A3.1* and *TaASN-D2* (Table 1). The most common presence/absence variation was of *TaASN-B2*, which was deleted in eight of the 15 genotypes assayed, including Chinese Spring (Table 1).

### Characterisation of the *TaASN-B2* deletion

**Fig. 1:**
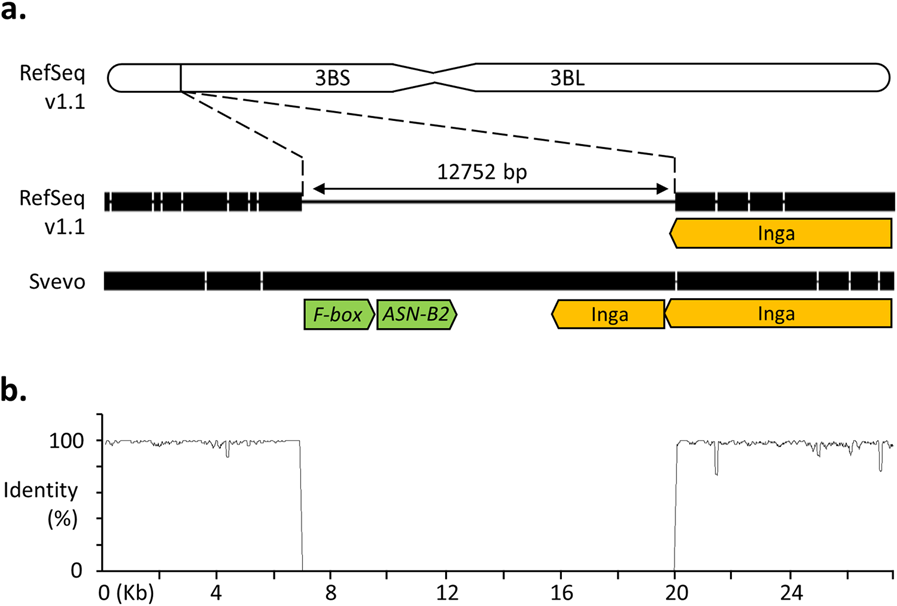
Variation at the *TaASN-B2* locus **a.** Diagram showing the location of the deletion in Chinese Spring and alignment with the corresponding region in variety Svevo. The deletion occurs at position 60,301,515 bp on chromosome 3B in the RefSeq v1.1 genome assembly. Notable gene and transposon annotations are shown. **b**. Plot of the nucleotide sequence identity (%) between Chinese Spring and Svevo in regions flanking the deletion, from approximately 7 kb upstream to 8 kb downstream. Sliding window average of 100 bp

The deletion containing *TaASN-B2* mapped to chromosome arm 3BS in the Chinese Spring RefSeq v1.1 genome assembly (Fig. 1a). Alignment of the surrounding region was performed between the annotated Chinese Spring genome and the corresponding region of the Svevo and Jagger genomes, both of which contain the *ASN-B2* gene, to evaluate other features of this locus (Fig. 1a and b). The deletion in Chinese Spring was 12,752 bp with respect to the Svevo genome and 12,770 bp with respect to the Jagger genome. A putative open reading frame predicted to encode an F-box protein was detected upstream of *TaASN-B2* in the deleted region (Fig. 1a). Directly downstream of the deletion in Chinese Spring and the corresponding region in Svevo and Jagger there is a large, long terminal repeat (LTR) retrotransposon, *Inga*, belonging to the Ty1-*copia* family [22] (Fig. 1a), the identity of which was confirmed using the TREP database [23]. Analysis of the other genome assemblies for the genotypes shown in Table 1 revealed that all eight varieties lacking *TaASN-B2* had identical breakpoints.

### Wider screening for the presence/absence of *ASN-B2*

**Fig. 2:**
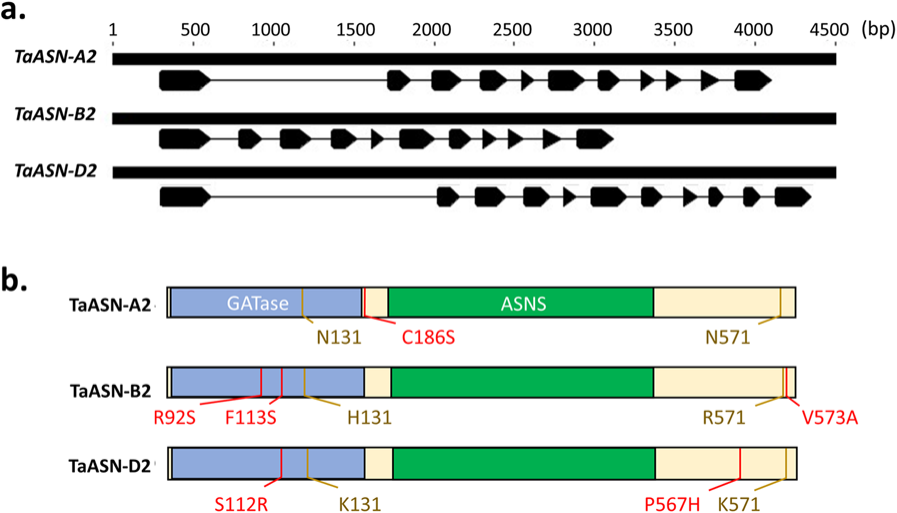
Structural characteristics of the *TaASN2* genes and proteins from Cadenza. **a**. Diagrammatic representation of the exon/intron structures of the *TaASN-A2*, *TaASN-B2* and *TaASN-D2* homeologues [15]. Open arrows indicate exons and lines indicate introns. **b**. Diagram representing the structure and similarity of the proteins encoded by each *TaASN2* homeologue, showing the glutamine amidotransferases (GATase) domain (amino acids approx. 2–185) and asparagine synthetase (ASNS) domain (amino acids approx. 210–450). Residues that differ across all three homeologues are highlighted in yellow, whereas residues that differ in a single homeologue are highlighted in red

Because of their potential role in determining free asparagine concentrations in the wheat grain, allelic variation in *ASN2* genes was explored in a broader set of wheat germplasm. Comparison of the three *TaASN2* homeologues in the Cadenza genome revealed they share a common gene structure, each containing 11 exons (Fig. 2a). The encoded proteins shared > 99% identity at the amino acid level, with only eight polymorphic residues between homeologues (Fig. 2b). Although four of these polymorphisms fell in the glutamine amidotransferase (GATase) domain (Fig. 2b), none were predicted to affect protein function according to the SIFT analysis (Table 1).

**Fig. 3:**
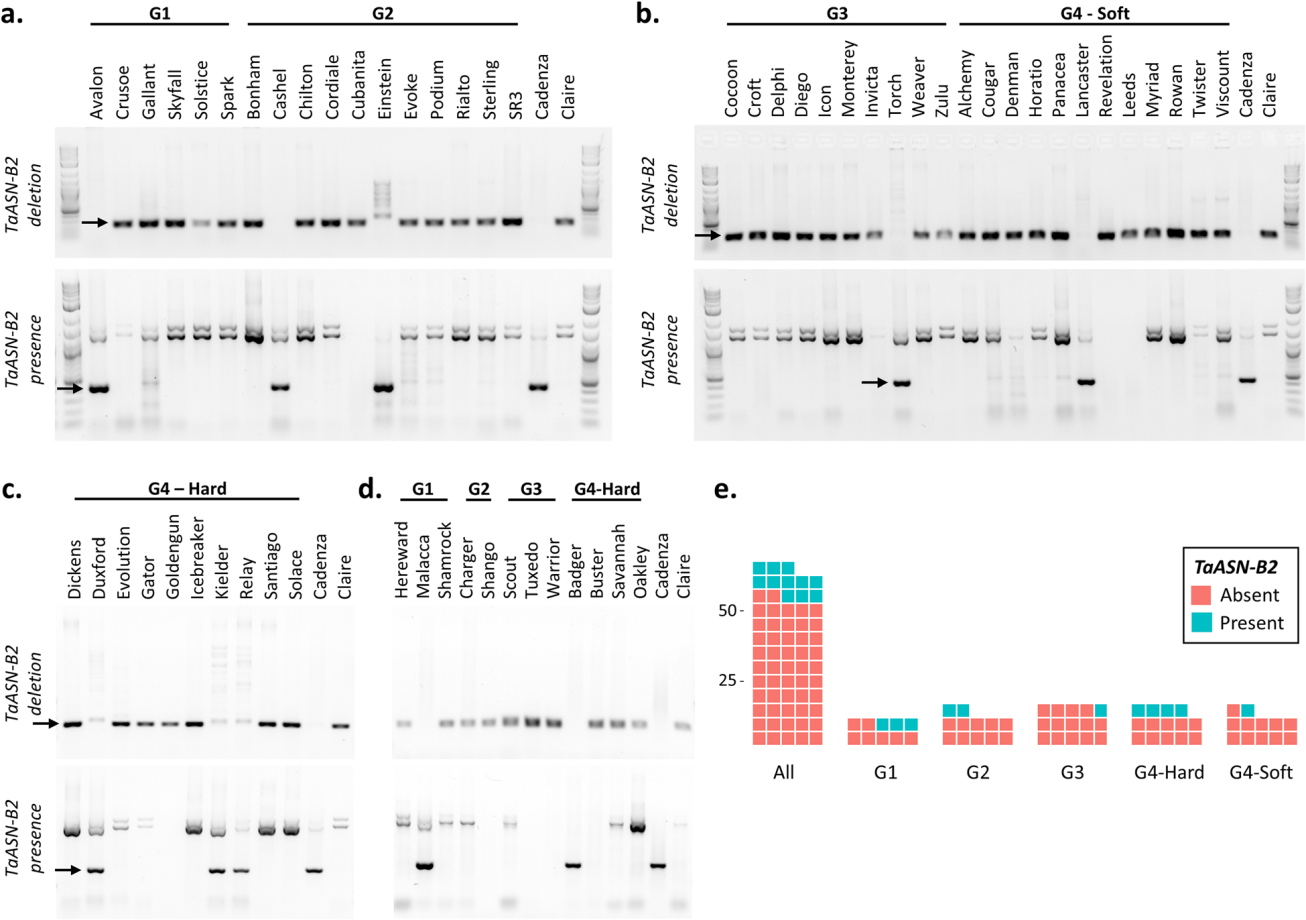
Presence/absence of *TaASN-B2* in UK wheat varieties. **a-d:** Electrophoresis gels of PCR products from assays to distinguish the presence and absence of *TaASN-B2* in a collection of UK wheat varieties grown across two years [11]. **a.** UK Flour Millers milling group 1 and 2 hard bread wheats (grown in 2012–2013). **b.** UK Flour Millers group 3 and 4 soft wheats (grown in 2012–2013). **c.** UK Flour Millers group 4 hard wheats (grown in 2012–2013). **d.** Remaining varieties grown only in 2011–2012. Varieties Cadenza and Claire were used as controls for *TaASN-B2* presence and absence, respectively. The distinguishing PCR products are indicated with arrows. **e.** Diagram showing the frequency of the *TaASN-B2* deletion in 63 UK wheat varieties, separated into UK Flour milling groups: G1 (breadmaking), G2 (breadmaking potential), G3 (soft/biscuit), G4 (feed/other)

The length of intron 1 varied between homeologues and was 1104 bp in *TaASN-A2*, 1411 bp in *TaASN-D2*, but only 175 bp in *TaASN-B2* (Fig. 2a). A pair of redundant primers was designed to amplify a DNA fragment from the first intron of all three homeologues, allowing for the reaction products to be readily distinguished based on size and to detect the presence of *TaASN-B2*. A second pair of homeologue-specific primers was designed to anneal upstream and downstream of the deleted region containing *TaASN-B2* to amplify a DNA fragment only in genotypes carrying this deletion. The presence of *TaASN-B2* was, therefore, demonstrated by the amplification of a 434 bp product with the first primer pair and failure to amplify a PCR product using the second primer pair. The results of the analysis are shown in Fig. 3a-d and summarised in Additional file 1, Table S2a. Overall, *TaASN-B2* was deleted in 52 of 63 UK winter wheat varieties assayed (82.5%) (Fig. 3e). The deletion was most common in the biscuit (G3) class (93.3%) and least common in the breadmaking (G1) (70%) class (Fig. 3e). An additional set of 24 global wheat varieties were analysed using a similar PCR assay (Additional file 1, Fig. S2a) and the results are shown in Additional file 1, Fig. S2b, and summarised in Additional file 1, Table S2b. The *TaASN-B2* deletion was less common among these wheats than in the UK varieties, being present in just 50% of the genotypes (Additional file 1, Table S2b).

**Fig. 4:**
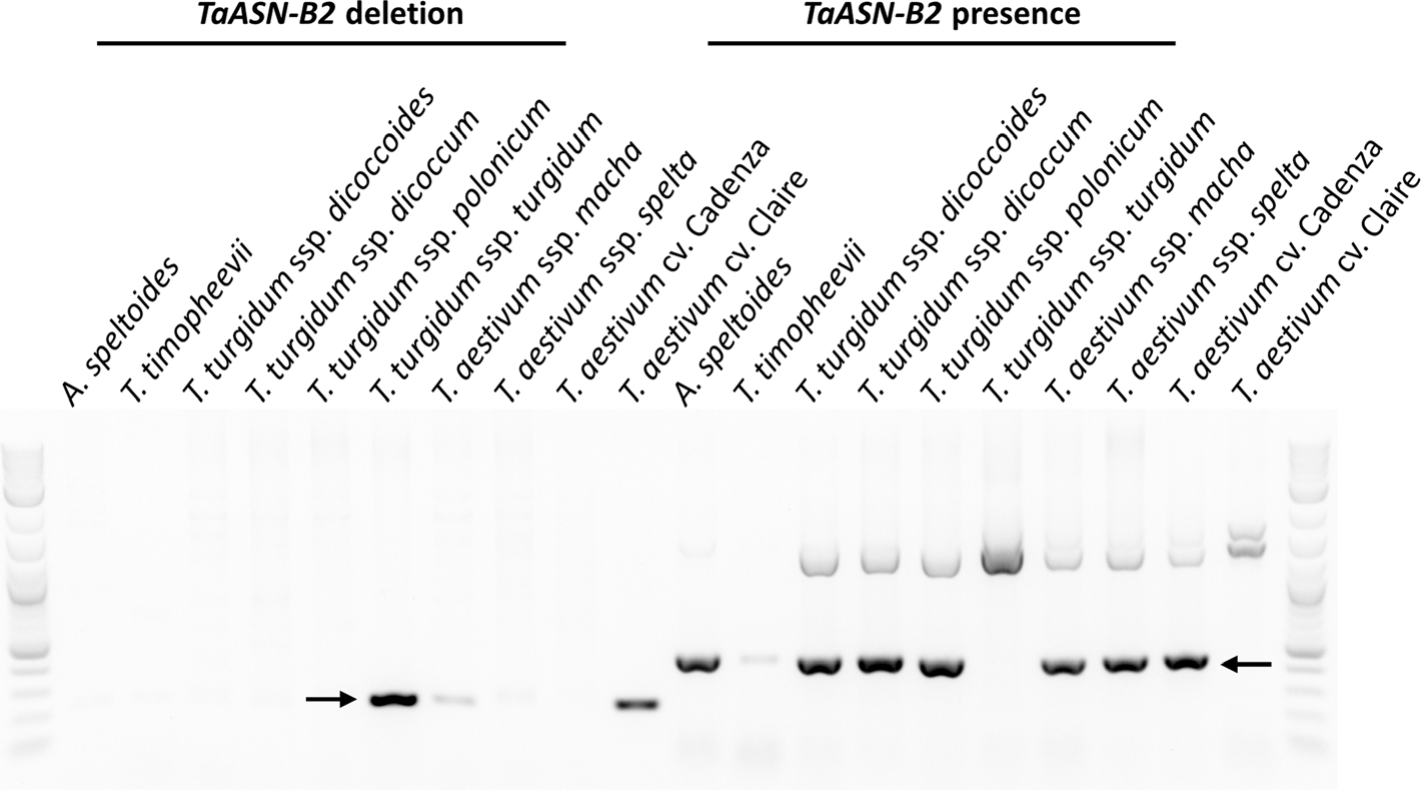
Presence/absence of *ASN-B2* in different wheat species. Electrophoresis gel of PCR products from assays to distinguish the presence and absence of *ASN-B2* in a selection of wheat species. *T. aestivum* varieties Cadenza and Claire were used as controls for *ASN-B2* presence and absence, respectively. The distinguishing PCR products are indicated with arrows

A selection of other wheat species was also screened for the presence of an *ASN-B2* gene (Fig. 4). An *ASN2* gene was identified in *Aegilops speltoides* (genome BB); however, while an *ASN-B2* gene was present in some tetraploid wheat genotypes (genomes AABB) it was absent in others (Fig. 4). Both pasta wheat (*T. turgidum* ssp. *durum)* varieties assayed in the study, Svevo and Kronos, were shown to have an *ASN-B2* gene, as was Polish wheat (*T. turgidum* ssp. *Polonicum*), but the gene was absent in rivet wheat (*T. turgidum* ssp. *turgidum*). There was some ambiguity in the result for makha wheat (*T. macha*) in that there was a clear positive result for the presence of the *ASN-B2* gene but a faint band amplified in the assay for the deletion (Fig. 4). This band was still present when the experiment was repeated (data not shown) and is likely due to genetic heterogeneity in the sample.

### Expression profiles of wheat *ASN* genes during development

**Fig. 5:**
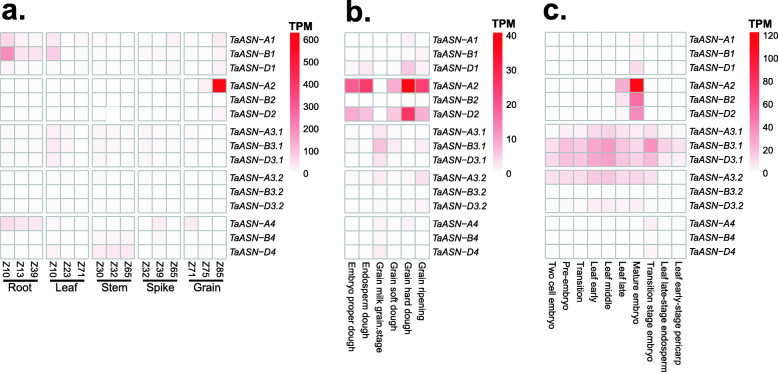
Expression profiles of *TaASN* genes in wheat derived from RNA-seq mapping data. **a.** Expression of *TaASN* genes in Chinese Spring across different tissues and developmental stages [24]. **b.** Expression of *TaASN* genes in grain tissues of the variety Azhurnaya across six stages of development [25]. **c.** Expression of *TaASN* genes in variety AC Barrie across an embryo development timecourse [26]

Raw sequencing reads from public RNA-seq datasets were mapped to the IWGSC RefSeq v1.1 genome assembly to provide a comprehensive overview of the expression profile of each *TaASN* gene. The expression values for each dataset are provided in Additional file 2 as mean Transcripts Per Million (TPM) values. In a dataset encompassing roots, leaves, stems, spike and grain, each sampled at three developmental stages [24], *TaASN1* transcript levels were highest in young roots and leaves, whereas the three homeologues of *TaASN3.1* and *TaASN3.2* showed a broader expression profile, with transcripts detected in all assayed tissue types across different stages of development (Fig. 5a). *TaASN4* homeologues were also broadly expressed, with *TaASN-A4* transcript levels highest in root and spike tissues, and *TaASN-B4* and *TaASN-D4* more highly expressed during stem development (Fig. 5a). As shown previously [18, 19], *TaASN2* showed a grain-specific expression profile, with transcript levels highest at Zadoks stage 85 (Z85), which corresponds to the soft dough stage [27] (Fig. 5a). Furthermore, *TaASN-A2* accounted for 83.3% of all *TaASN* transcripts in grain tissues at Z85, while *TaASN-D2* contributed just 3.0%, consistent with previous results [19].

To further explore the expression of *TaASN* genes in the grain, an expression dataset from six stages of grain development in the variety Azhurnaya was analysed [25]. As expected [18, 19], high transcript levels of *TaASN-A2* and *TaASN-D2* were found in five developmental stages which, when combined, accounted for between 69 and 86% of all *TaASN* transcripts in these tissues (Fig. 5b). The exception was the grain milk stage, where *TaASN3.1* transcript levels were higher than *TaASN2* (Fig. 5b), as shown previously [18]. *TaASN-B2* transcripts were detected at negligible levels in this dataset, suggesting its deletion in the Azhurnaya genome.

Analysis of expression data from an embryo development timecourse in the common wheat variety AC Barrie [28] revealed that *TaASN2* transcript levels were highest in the mature embryo stage (Fig. 5c). Among *TaASN2* homeologues, *TaASN-A2* was again the most highly expressed gene, while *TaASN-B2* and *TaASN-D2* were expressed at similar levels (Fig. 5c). However, *TaASN2* transcripts were detected only at negligible levels at all other developmental timepoints, including earlier stages of embryo development and in endosperm and pericarp tissues, where *TaASN3.1* transcripts were more abundant (Fig. 5c). Taken together, these data confirm the specific activity of *TaASN2* in grain tissues and the mature embryo, and indicate a broader role for *TaASN3.1* genes across development, including the early stages of embryo development.

### Inter-varietal variation in *TaASN* expression profiles during grain development

**Fig. 6:**
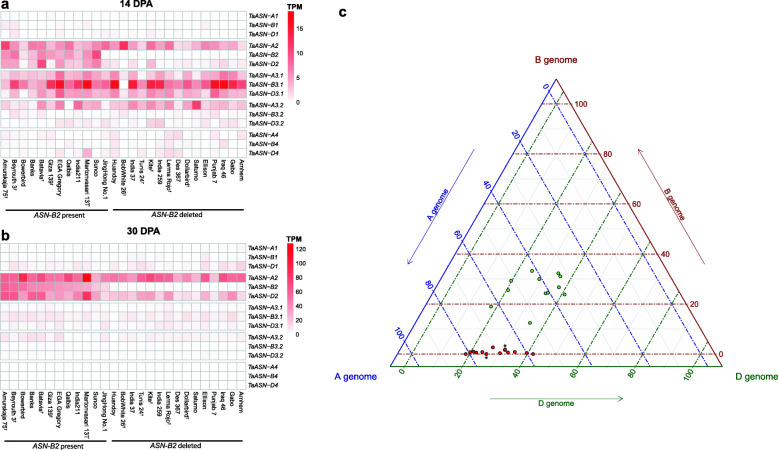
Expression of *TaASN* genes in 27 worldwide wheat varieties from RNA-seq mapping data [28]. **a.** Grain tissues 14 DPA. **b.** Grain tissues 30 DPA. † indicates varieties for which the presence or absence of *TaASN-B2* was confirmed by PCR **c.** Ternary plot illustrating the relative contribution of A, B and D genome *TaASN2* homeologues to total *TaASN2* gene expression in grain tissues at 30 DPA in each variety. Varieties with *TaASN-B2* present are indicated with green circles, varieties with *TaASN-B2* deleted are indicated with red circles

To analyse variation in *ASN* transcript levels in wheat grain, RNA-seq reads were mapped from grain samples at 14 days post anthesis (DPA) and 30 DPA taken from 27 worldwide wheat varieties [28]. At 14 DPA, total *TaASN3.1* transcript levels ranged from 2 to 32 TPM and were greater than *TaASN2* in 22 of the 27 varieties assayed (Fig. 6a), consistent with previous results [19]. At 30 DPA, *TaASN2* homeologues were the most highly expressed asparagine synthetase genes in all varieties assayed (Fig. 6b). At this latter timepoint, total *TaASN2* transcript levels showed large variation between genotypes, ranging from 28 to 242 TPM (Additional file 2). Several lines exhibited very low *TaASN-B2* transcript levels and the deletion of this gene was confirmed in five of these lines using the PCR assay (Additional file 1, Fig. S2b; Additional file 1, Table S2b). There were also lines with readily detectable *TaASN-B2* transcripts (>five TPM), and the presence of this gene was confirmed for four of these lines (Additional file 1, Fig. S2b; Additional file 1, Table S2b).

A ternary plot showing the relative contributions of each homeologue to overall *TaASN2* transcript levels in the grain at 30 DPA in different wheat varieties (Fig. 6c) revealed that *TaASN-A2* transcript levels were generally greater than *TaASN-D2* in varieties with very low *TaASN-B2* transcript levels (likely associated with the deletion of this gene in these varieties). By contrast, in varieties with relatively high *TaASN-B2* expression, *TaASN-A2* and *TaASN-D2* were generally more evenly expressed, and in four varieties, *TaASN-D2* transcript levels were higher than *TaASN-A2* (Fig. 6c). Nevertheless, overall TPM values for *TaASN-A2* and *TaASN-D2* were not higher in varieties that lacked *TaASN-B2* compared with those in which *TaASN-B2* was present (Additional file 2), so there was no evidence of increased expression of these genes to compensate for the lack of *TaASN-B2* transcripts.

**Fig. 7:**
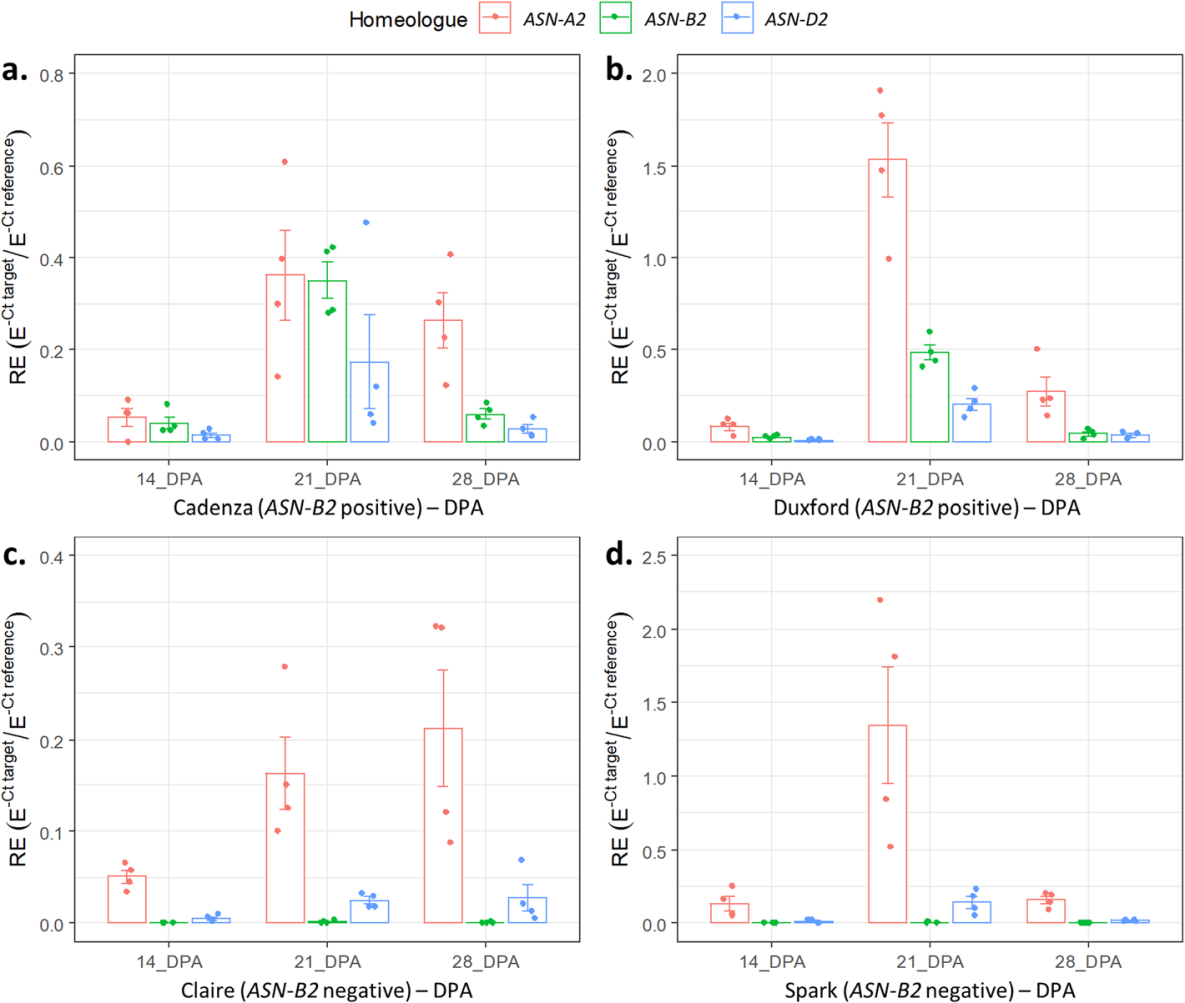
Expression analysis by RT-qPCR of the *TaASN2* homeologues in the embryo of four varieties of wheat either possessing the *TaASN-B2* gene (Cadenza (**a.**) and Duxford (**b.**)) or lacking one (Claire (**c.**) and Spark (**d.**)) at 14-, 21-, and 28-days post anthesis (DPA). Expression levels are relative to three reference genes (*GAPDH*, *PROSM*, and *SDH*). RE (relative expression), E (PCR efficiency calculated by LinRegPCR), Ct (threshold cycle). Error bars show standard error of the mean

To investigate the expression dynamics further, the expression of the *TaASN2* homeologues was also analysed by RT-qPCR in two wheat varieties possessing *TaASN-B2* (Cadenza and Duxford) and two varieties lacking it (Spark and Claire) (Fig. 7). The results of the analysis of variance for this experiment are shown in Additional file 1, Table S3a, revealing significant effects (*p* < 0.001) of variety, timepoint, and homeologue, and the interactions between these factors, on relative expression levels. In Cadenza and Duxford, mean *TaASN-A2* expression was the highest of the three homeologues across all timepoints (14, 21 and 28 DPA), whereas mean *TaASN-D2* expression was the lowest (Fig. 7a and b). In both varieties, mean *TaASN-B2* expression was greater than *TaASN-D2* expression at all timepoints, and in Cadenza at 21 DPA, matched the levels of *TaASN-A2* expression (Fig. 7a). In Claire and Spark, mean *TaASN-A2* expression was greater than *TaASN-D2* expression in all samples, and showed similar expression dynamics across timepoints to Cadenza and Duxford (Fig. 7c and d). Notably, there was no evidence of higher expression of *TaASN-A2* or *TaASN-D2* in Claire and Spark compared with Cadenza and Duxford.

### Contribution of *TaASN-B2* to free asparagine concentration in the grain

The screen of varieties for the presence or absence of *TaASN-B2* (Fig. 3, Additional file 1, Additional file 1, Table S2) included 63 UK varieties for which free asparagine concentration in the grain had been determined in field trials grown in the UK over two growing seasons (2011–2012 and 2012–2013) [11]. This meant that an assessment could be made of the effect of the *TaASN-B2* deletion on free asparagine concentrations in the grain. Of the 63 varieties in the field trials, eleven possessed *TaASN-B2* while 52 did not.

**Fig. 8:**
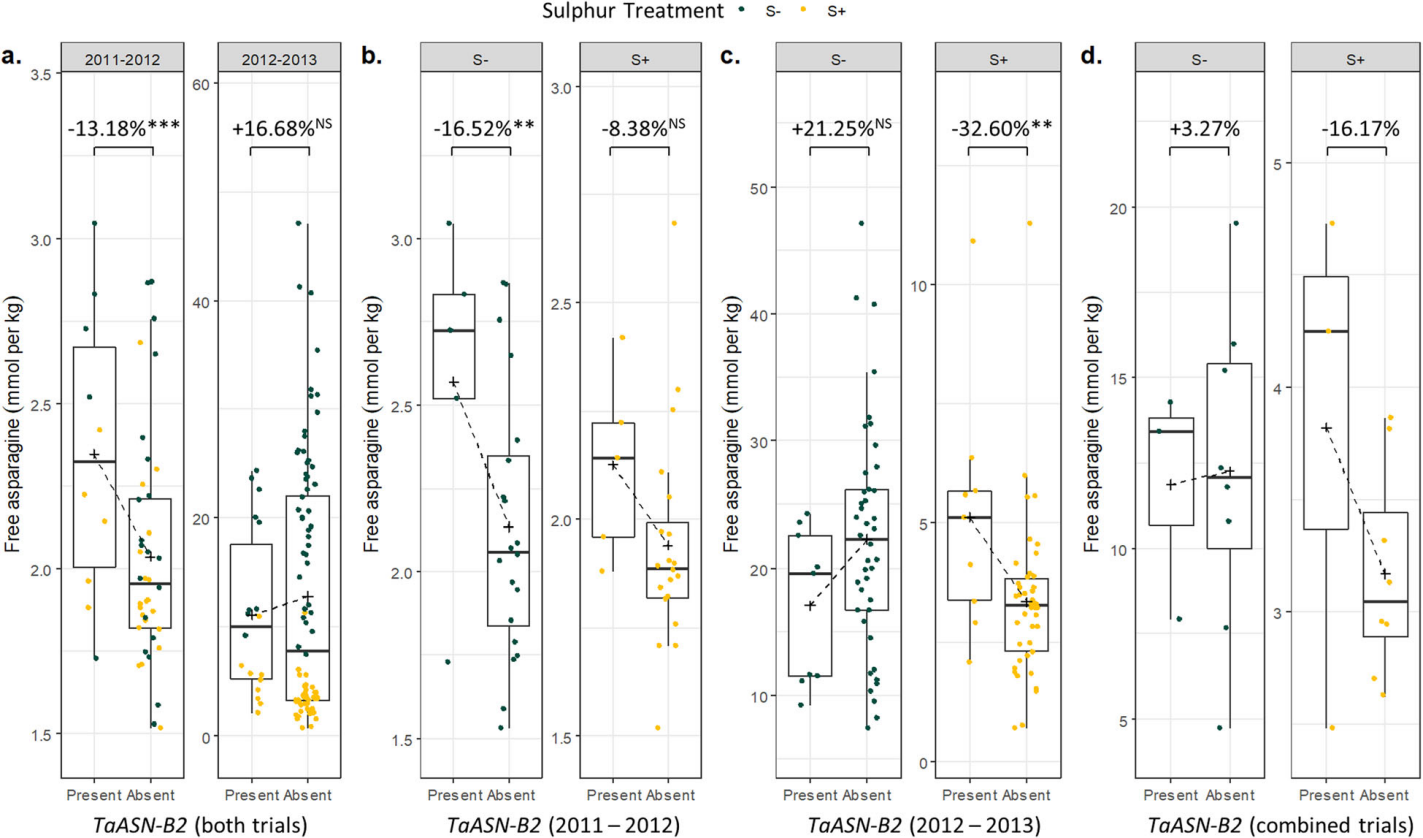
Concentrations of free asparagine in the grain of different varieties of wheat in which the *TaASN-B2* gene was either present or absent in two field trials, 2011–2012 and 2012–2013, showing the effect of the *TaASN-B2* deletion under different sulphur treatments (S- and S+) and the interaction between the presence/absence of the gene and treatment. (a) Back-transformed data from the 2011–2012 and 2012–2013 trials with concentrations from the S- and S+ treatments plotted together. (b) Back-transformed data from the 2011–2012 season with concentrations from the S- and S+ treatments plotted separately. (c) Back-transformed data from the 2012–2013 season with concentrations from the S- and S+ treatments plotted separately. (d) Back-transformed predicted means from the REML analysis for the 11 varieties grown in both trials. Crosses indicate the mean of each group. Boxes show the interquartile range and median. Whiskers show the smallest and largest value within 1.5 times of the interquartile range above and below the 75th and 25th percentile, respectively. Significance scores for the main effect of *TaASN-B2* in (a) were taken from separate ANOVA analyses performed on log_*e*_ transformed data. Multiple comparisons with Bonferroni correction were performed following the ANOVA analyses for *TaASN-B2* group comparisons in 2011–2012 (b) and 2012–2013 (c). NS = *p* > 0.05, ** = *p* < 0.01, *** = *p* < 0.001

The grain from these field trials had been produced in plots in which sulphur was either supplied or withheld [11]. We analysed the effect of *TaASN-B2* alongside the other variables in these trials by ANOVA (Additional file 1, Table S3b), which revealed a significant (*p* < 0.001) effect of the *TaASN-B2* deletion in the 2011–2012 field trial: varieties without *TaASN-B2* had 13.18% less free asparagine relative to those with *TaASN-B2* (Fig. 8a). There was no significant effect (*p* > 0.05) of the deletion by itself in the 2012–2013 field trial, but there was a significant (*p* < 0.001) interaction between *TaASN-B2* presence/absence and sulphur treatment: there was no significant difference (*p* > 0.05) in free asparagine concentrations between varieties with and without *TaASN-B2* under sulphur deficiency, but varieties without *TaASN-B2* had 32.60% less free asparagine (*p* < 0.01) than those with *TaASN-B2* under sulphur sufficiency (Fig. 8c).

The REML analysis based on the complete combined datasets also identified a significant effect of *TaASN-B2* as well as an interaction between *TaASN-B2* and sulphur treatment (Additional file 1, Table S3b). This analysis also further demonstrated that the interaction between *TaASN-B2* and sulphur treatment changed by year, as is suggested by Fig. 8b and c, and that there is a significant interaction between year and sulphur treatment. Prediction and analysis of means from the REML analysis for the 11 varieties common to both trials (of the 63 that were analysed in total) suggests that the *TaASN-B2* deletion effect is greatest under sulphur sufficiency across different growing seasons (Fig. 8d). The effect of sulphur deficiency differed greatly between the trials though, so it is difficult to reach a definitive conclusion regarding the effect of *TaASN-B2* under sulphur deficiency based on the predicted means of the REML analysis.

## Discussion

### A natural deletion of *ASN-B2* in wheat

In this study, wheat genomic resources were utilised to characterise a natural deletion that includes the complete *TaASN-B2* gene and to design a molecular assay to trace its frequency in a diverse set of wheat germplasm. The break points for this deletion event were identical in all eight common wheat genome assemblies that were analysed, strongly suggesting a single, common origin for this allele. The deleted region is in close proximity to a Ty1-*copia* transposable element (Fig. 1a), one of the most abundant classes of LTR retrotransposons in the wheat genome [29], and a potential causative agent for the deletion. The *ASN-B2* gene was intact in the B genome of *Ae. speltoides* (genome BB) (Fig. 4) and this genome is related to the B genome progenitor of domesticated wheat species. However, the direct ancestor of the hybridisation event originating emmer wheat (genomes AABB) has yet to be identified [16], so firm conclusions of the origins of the *ASN-B2* deletion in diploid wheat progenitors cannot be drawn. Some wild and domesticated tetraploid wheats carried an intact *ASN-B2* gene whereas in others it was absent, which, as discussed elsewhere [14, 17], suggests the occurrence of independent hybridisation events in wheat’s evolutionary history. Studying haplotype variation at this locus in more diverse wheat germplasm collections, including more wild and domesticated emmer wheats from different subpopulations within the fertile crescent [16], could shed light on the origins of the *ASN-B2* deletion.

Among different classes of UK winter common wheat varieties, this deletion was found at high frequencies (Fig. 3e). The unbalanced distribution of this allele could result from the lack of genetic diversity during early selections in UK wheat variety development, be an artefact of the selection of varieties analysed in this study, or simply have occurred by chance, indicating that this allele is under neutral selection. However, it is also possible that this allele is subject to direct or indirect selection due to a positive impact on plant fitness or performance. If so, it is unlikely to have been selected due to its association with free asparagine content in the grain described in the current study, because selections based on asparagine content have been made only very recently, if at all. Based on the grain-specific expression profile of *TaASN-B2*, other possible traits that might account for positive selection of the deletion are pre-harvest sprouting resistance or other quality traits shown in some studies to be correlated with asparagine concentrations [30]. However, other studies have found no association between asparagine content and a host of baking quality traits [12], so this association requires continued analysis. A further possibility is that this deletion has been selected indirectly due to a beneficial genetic variant in linkage disequilibrium with this locus. Evidence of marker-trait associations for other agronomic traits in this region would support this hypothesis, although it would be difficult to test directly. A more detailed functional characterisation of the *ASN2* genes focused on grain development and quality traits may reveal additional, previously unidentified roles of this gene as well; similar to the moonlighting roles found for asparagine synthetase in yeast and mammalian cells [31, 32].

### Expression profiles of wheat asparagine synthetase genes

Our expression data confirmed previous findings [18, 19] that, in many varieties, *TaASN-A2* is the most highly expressed *TaASN2* homeologue during grain development (Figs. 5, 6 and 7). In five of the 12 varieties possessing *TaASN-B2* that have been assayed by RNA-seq, *TaASN-B2* contributes a higher proportion of *ASN2* transcripts than *TaASN-D2*, whereas *TaASN-D2* expression is greater in the other seven varieties (Fig. 6c, Additional file 2). Higher *TaASN-B2* expression over *TaASN-D2* was also observed in varieties Cadenza and Duxford via RT-qPCR, throughout the sampling time course from 14 to 28 DPA (Fig. 7). In every variety lacking *TaASN-B2*, *TaASN-A2* contributed a greater proportion of *ASN2* transcripts than *TaASN-D2* (Fig. 6c). By contrast, in varieties with *TaASN-B2* present the three homeologues were more evenly expressed, although *TaASN-A2* still contributed the greatest proportion of *ASN2* transcripts in most lines (Fig. 6c). However, based on our combined RNA-seq and RT-qPCR data, the loss of *TaASN-B2* is associated with an overall reduction of *ASN2* transcript levels and no compensatory upregulation of the A or D homeologues, which is consistent with the effect of this deletion on grain asparagine concentration. This is in contrast to other biosynthetic pathways, for example in GA signalling [33], in which feedback mechanisms modulate the transcript levels of biosynthetic genes to compensate for loss of expression.

Our expression analyses also provide additional insight into the potential role of *TaASN3.1* during embryo and grain development. Transcript levels of this gene were most abundant at earlier stages of grain development, while *TaASN2* transcripts predominated later in grain development (Figs. 5 and 6). In most, but not all, varieties, expression of *TaASN3.1* remained higher than *TaASN2* even by 14 DPA, in contrast to a previous study in which *TaASN2* was found to be the most highly expressed asparagine synthetase gene in the embryo and endosperm of the common wheat variety Spark and doubled haploid line SR3 at this developmental stage [18, 19]. This is most likely explained by differences in growth conditions and developmental rates; indeed, differential rates of development and asparagine synthetase gene expression were observed between Spark and SR3 even under identical growth conditions [19]. Therefore, it would be interesting to characterise the function of *TaASN3.1* in wheat to help understand the extent to which this gene contributes to asparagine biosynthesis in the grain, and its importance for grain development. Allelic variation in this gene could also contribute to reduced free asparagine content in the grain, and it would be possible to test this hypothesis either by characterizing the effect of potentially disruptive natural *TaASN-A3.1* alleles carried by some varieties (Table 1), or by targeted mutagenesis. This approach could also be applied to characterise the function of other wheat asparagine synthetase genes, to help assess the possibility of integrating genetic variation in this family into wheat breeding programmes.

### Breeding for reduced grain asparagine content

The *TaASN-B2* deletion was associated with a 13.18% reduction in grain free asparagine concentration in the 2011–2012 field trial (Fig. 8a) and with a 32.60% reduction under sulphur sufficiency in the 2012–2013 field trial (Fig. 8c), showing that this variant can contribute to reduced grain free asparagine concentrations in field conditions. The milder effect of sulphur deficiency on free asparagine concentrations in 2011–2012 was previously noted [11] and is likely why the effect of *TaASN-B2* was seen across both sulphur treatments. In contrast, sulphur deficiency caused a much greater increase in free asparagine concentrations in the 2012–2013 trial, bringing about an interaction between sulphur treatment and *TaASN-B2* (Additional file 1, Table S3b) and demonstrating that the deletion does not have an effect under severe sulphur deficiency when free asparagine levels are higher (Fig. 8c). This is consistent with earlier studies showing strong environmental (E) as well as genetic (G) impacts on free asparagine content, as well as complex G × E interactions [3]. It implies that the effect of the *TaASN-B2* deletion can be maximised by employing appropriate crop management strategies to reduce stress.

This retrospective analysis of the *TaASN-B2* deletion on grain free asparagine levels was limited because varieties in the trials were not originally selected based on the presence or absence of *TaASN-B2*, so there were many more varieties without *TaASN-B2* than those with it in this analysis. Consequently, it will be important to characterise the impact of the *TaASN-B2* deletion in replicated field trials in additional and more diverse environments, including phenotyping a broader set of traits to confirm that there are no detrimental pleiotropic effects associated with this allele. To minimise the impact of other genetic variation, these trials could be performed in a common genetic background, either by developing near isogenic lines, or by directly inducing genetic variation by mutagenesis.

Measuring free asparagine concentrations in grain directly is expensive, requires specialist analytical equipment and is often impractical for breeders, but the use of our simple PCR screen could enable wheat breeders to exclude genotypes that are more likely to have high free asparagine concentrations in the grain when grown in sulphur-sufficient conditions (Fig. 8b). Although the effect is mild, this could contribute to reducing the public health risk associated with dietary acrylamide and help food manufacturers to comply with the difficult and evolving regulations on the presence of acrylamide in their products. This deletion can be fully exploited in all regulatory environments due to its natural origins, in the same way that other quality traits have been exploited in different crop species. For example, some barley genotypes possess reduced cadmium accumulation due to the natural insertion of a transposable element upstream of a cadmium transporter, and the allele carrying this insertion can be used without restriction [34].

Although the impact of this specific variant may be limited for UK wheat breeders because of its high frequency in UK winter wheat varieties (Fig. 3e, Additional file 1, Table S2a), there may be greater opportunity to apply this allele in other regions of the world, since just 50% of wheat varieties from a selection of global varieties carried the deletion (Additional file 1, Table S2b). This panel included varieties from Australia, Africa and Europe, so it would be worthwhile exploring the frequency of this deletion in broader collections of wheat germplasm. Although only two durum wheat varieties were included in the current study, both carried the *ASN-B2* gene, possibly indicating that the historic hybridisation events giving rise to durum wheats may have included the *ASN-B2* gene at higher frequencies than for common wheat. Therefore, durum wheat breeders may have an opportunity to reduce free asparagine concentration in the grain by identifying and selecting genotypes carrying this deletion. Furthermore, because the durum wheat genome is tetraploid, those varieties lacking the *ASN-B2* homeologue may show a proportionally greater reduction in grain asparagine than that found in hexaploid common wheat. A major use of durum wheat is for pasta production and although acrylamide is present in pasta, it is at relatively low levels [35]. However, durum wheat grain is also incorporated into grists for making products in which acrylamide levels are likely to be higher, such as pizza bases, pitta bread and other flatbreads.

Although we detected only two different *TaASN-D2* alleles (one only found in the variety SY Mattis) and no variation in *TaASN-A2* (Table 1), it is possible that broader screens of more diverse germplasm may yield additional natural variants that could be integrated into breeding programmes to select for reduced free asparagine concentration. Previous association mapping studies have identified QTL controlling asparagine content [12, 13], but these QTL did not map to regions of the genome containing asparagine synthetase genes. However, reverse genetics tools, such as EMS- or CRISPR/Cas9-induced mutagenesis, provide the potential to engineer allelic diversity that does not exist among wheat germplasm, including combinations of recessive mutations that are unlikely to be selected due to functional redundancy in polyploid genomes [36, 37]. The power of this approach was demonstrated in a recent study in which plants exhibiting reductions in free asparagine concentrations of up to 90% were developed by editing all six *ASN2* alleles using CRISPR/Cas9 [20]. Furthermore, the presence of three homeologues of this gene allows for selection of combinations of allelic knockouts that may allow breeders to balance reduced free asparagine content with other grain development traits. Although this would be a powerful and rapid approach to engineer and characterise potentially valuable genetic variation, it is important to note the complex and dynamic regulatory landscape that currently restricts applications of CRISPR-Cas9 in plant breeding in some regions of the world [38].

## Conclusions

Characterisation of natural allelic variation in the wheat asparagine synthetase gene family identified a deletion of just under 13 kb encompassing *TaASN-B2* that is present at high frequencies among UK winter wheat varieties. The deletion was also present in some wild emmer wheats, suggesting its ancient origins and retention during domestication and modern breeding. The allele carrying the deletion was associated with a reduction in free asparagine content in field experiments and could be selected using an inexpensive PCR assay to help breeders develop low-asparagine wheat varieties.

## Methods

### Genomic analyses

Nucleotide sequence data for the wheat *ASN* genes from different wheat genotypes were obtained using the BLAST tools of the 10+ Wheat Genomes Project (https://webblast.ipk-gatersleben.de/wheat_ten_genomes/), the Grassroots Genomics Project (https://wheatis.tgac.ac.uk/grassroots-portal/blast) [39], and the Graingenes database (https://wheat.pw.usda.gov/cgi-bin/seqserve/blast_wheat.cgi) [40]. Some *ASN* genes lacked complete sequence information, and these exceptions are described in Additional file 1, Table S4). Geneious Prime 2020.1.2 was used for alignments and sequence identity analyses between genes. The annotated genome from the pasta wheat (*T. durum*) variety Svevo (https://www.interomics.eu/durum-wheat-genome) [41] was used to compare the genomic region containing *TaASN-B2* with the corresponding region in the RefSeq v1.1 genome from the common wheat (*T. aestivum* L.) landrace Chinese Spring (from Ensembl plants https://plants.ensembl.org/wheat) [21]. The softberry-FGENESH tool [42] was used to identify putative genes from the 12,770 bp deleted region encompassing *TaASN-B2* from the variety Jagger. This analysis identified five putative ORFs. Each was analysed with HMMScan [43] using an e-value cutoff of 0.05, which confirmed the presence of *TaASN-B2* and a second gene encoding a protein containing an F-box PFam domain (PF00646). Transposon annotations were confirmed using the BLAST tool in TREP (TRansposable Elements Platform) (http://botserv2.uzh.ch/kelldata/trep-db/index.html) [23]. SIFT analysis [44] was performed by comparing each ASN protein from Chinese Spring with the protein encoded by the orthologous gene from other wheat varieties. For *TaASN-B2*, the protein from Jagger was used as a reference.

### Plant materials and germination

Seeds of UK cultivars were either maintained at Rothamsted Research or obtained from stocks produced in the field trials studied here [11]. Other wheat varieties were obtained from USDA-ARS National Small Grains Collection (https://www.ars.usda.gov/) and the Germplasm Resource Unit at the John Innes Centre (www.seedstor.ac.uk). The names of all varieties included in this study are listed in Additional file 1, Table S2. Seed surface sterilisation was performed by incubating seeds in 70% ethanol for 10 min and then in 20% (v/v) sodium hypochlorite solution for 60 min with gentle agitation to ensure homogenous sterilisation of the seeds. Seeds were subsequently washed four times with sterile distilled water and left to germinate under continuous light at room temperature in sterile 90 mm Petri dishes on wet filter paper, sealed with Parafilm (Fisher Scientific Ltd., Loughborough, UK). For older seeds, plates were wrapped in foil and incubated at 4 °C for two to seven days to break dormancy, before transferring to continuous light and room temperature for germination. For seeds unable to germinate using either method, the embryo was dissected and placed in 90 mm Petri dishes containing MS media (4.4 g/L MS salts [45], 3% sucrose (30 g/L), pH 5.8, 7 g/L agar). These plates were then sealed with Parafilm and left to germinate at room temperature and continuous light.

### DNA extraction

DNA was extracted from leaf material of seedlings using the Wizard® Genomic DNA Purification Kit according to the manufacturer’s instructions (Promega (UK) Ltd., Southampton, UK). For seeds that failed to germinate using the above methods, embryos were dissected from multiple seeds and ground together into a fine powder for DNA extraction by the CTAB method [46]. DNA quality and abundance were assayed using a NanoDrop™ 1000 Spectrophotometer (Thermo Fisher Scientific).

### PCR assays to detect *TaASN-B2*

Homeologue-specific primers ASN-B2-Deletion-F and ASN-B2-Deletion-R (Additional file 1, Table S5) were designed to anneal upstream and downstream of the deletion site so that the absence of *TaASN-B2* could be demonstrated as a positive result with the amplification of a 232 bp DNA fragment. Another pair of primers to amplify the first intron of all *TaASN2* homeologues, ASN-2-Universal-F and ASN-2-Universal-R (Additional file 1, Table S5), was designed to test for and distinguish the presence or absence of all three *TaASN2* homeologues based on size. These forward and reverse primers amplified DNA fragments of sizes 1363 bp, 434 bp and 1670 bp, corresponding to *TaASN-A2*, *TaASN-B2* and *TaASN-D2*, respectively. These two primer sets were used in combination to verify the presence or absence of *ASN-B2.*

These primers were used in PCR reactions in volumes of 25 μL using 1 × DreamTaq™ PCR Master Mix (1.5 mM MgCl_2_) (Thermo Fisher Scientific, Epsom, UK) and including 1 μM of each primer and 50–150 ng of genomic DNA. Cycling conditions were identical for both primer sets: 5 min denaturation at 96 °C; 32 cycles of 30 s denaturation at 96 °C, 30 s annealing at 60.5 °C, 1.5 min extension at 72 °C; 10 min final extension at 72 °C. Reactions were analysed by electrophoresis on an agarose gel (1.0% w/v, ethidium bromide staining) with 1 kb Plus DNA Ladder (NEB, UK) and visualised using UV light in the Geldoc imaging system (BioRad, USA).

A separate PCR assay was used to confirm the presence or absence of *ASN-B2* in a set of 24 global wheat varieties (Additional file 1, Fig. S2). This assay used two sets of primers in a single PCR to amplify different amplicons depending on the presence or absence of *ASN-B2*. The primers used were ASN-B2_qF1 (P3) and ASN-B2_qR1 (P4), which amplify a DNA fragment of 125 bp in varieties carrying *TaASN-B2*, along with ASN-B2_CS_F3 (P1) and ASN-B2_CS_R1 (P2), which amplify a DNA fragment of 189 bp in varieties lacking the *TaASN-B2* gene (Additional file 1, Fig. S2). The PCR mixture included, in a total volume of 25 μL, 0.2 μM of primers P1 and P2 and 0.24 μM of primers P3 and P4, 1 × Standard *Taq* buffer, 250 ng template DNA and 0.125 μL *Taq* polymerase (New England Biolabs, Ipswich, MA, USA). Amplification was carried out using the following conditions: 95 °C for 30 s; 35 cycles of: 95 °C for 15 s, 59 °C for 30 s, 68 °C for 30 s; 68 °C for 5 min. Amplified DNA fragments were separated by electrophoresis on a 3% agarose gel stained with SYBR Safe (ApexBio, Houston, TX, USA). A single amplified DNA fragment of either 189 bp or 125 bp was expected from each reaction (Additional file 1, Fig. S2b). Full, uncropped images of all electrophoresis gels are provided in Additional file 3.

### RT-qPCR

Two common wheat varieties carrying *TaASN-B2* (Cadenza and Duxford) and two lacking the gene (Claire and Spark) were grown in a randomised block design in a glasshouse. Plants were grown in individual pots for destructive sampling and four replicates were taken at each timepoint. RNA was extracted from embryo tissue at three timepoints (14-, 21-, and 28-days post anthesis) using a standardised RNA extraction protocol [47]. The RNA was cleaned further using the ReliaPrep™ RNA Clean-Up and Concentration System (Promega) according to the manufacturer’s instructions. DNA was then removed from these samples using RQ1 RNase-Free DNase (Promega) according to the manufacturer’s instructions, and the RNA was quantified using a NanoDrop™ 1000 Spectrophotometer (Thermo Fisher Scientific).

cDNA was synthesised by mixing 2 μg RNA in nuclease-free water with oligo-dT primer, dNTPs, and SuperScript™ III Reverse Transcriptase kit components (Invitrogen), according to the manufacturer’s instructions, and placing in a thermocycler using the following programme: 95 °C for 5 min; 60 °C for 60 min; 72 °C for 15 min.

RT-qPCR was performed using an Applied Biosystems™ 7500 Real-Time PCR System set to ddCt (relative quantitation) mode. Each reaction contained 10 μL SYBR Green Master Mix (Applied Biosystems), 5 μL primer mastermix (containing 0.04 mM of each primer and ROX reference dye in nuclease-free water), and 5 μL cDNA (diluted to 6 ng/μL). The expression of each target gene was measured relative to three reference genes; *GAPDH*, *PROSM*, and *SDH*. Details of the primers used are found in Additional file 1, Table S5.

Relative expression values were calculated as described by Rieu and Powers [48]. Applied Biosystems 7500 Real-Time PCR Software version 2.0.5 was used to calculate Ct values and exported Rn data were converted to PCR efficiency data using LinRegPCR [49]. Statistical tests were performed using GenStat [50] to account for the blocking structure of the experiment and graphs were plotted in R [51] using the package ggpubr [52].

### RNA-seq data analysis

Raw RNA-seq reads from a developmental timecourse in the landrace Chinese Spring [24], grain development samples from the variety Azhurnaya [25], an embryo development timecourse from the variety AC Barrie [53] and grain expression at 14 DPA and 30 DPA from a set of worldwide wheat varieties [28], were downloaded from the NCBI GEO database (https://www.ncbi.nlm.nih.gov/geo/) processed and mapped to the IWGSC RefSeq v1.1 genome following the approach described previously [54]. An additional contig corresponding to the *TaASN-B2* coding sequence and including 1 kb of sequence upstream and downstream of the protein coding region was added to the reference genome to assay transcript levels of this gene. Raw counts were converted into TPM using a custom python script. Heatmaps were generated in R (v1.12.5019) using the command ‘heatmap’ within the gplots package [55]. The ternary plot was created using the ggtern package [26] within ggplot2 [56]. All expression data in TPM are presented in Additional file 2.

### Effect of *TaASN-B2* on free asparagine in the grain

Data from field trials performed in 2011–2012 and 2012–2013 [11] were used to investigate the effect of the *TaASN-B2* deletion on grain asparagine levels with sulphur either supplied (S+) or withheld (S-). Varieties from this field trial were screened as described above for the presence or absence of *TaASN-B2*. Data were log_e_ transformed to account for heterogeneity of variance, as identified in the previous study [11], before performing the ANOVA and REML analyses. Analyses and plotting were performed in R [51] with the package ggpubr [52]. ANOVA and REML analyses were performed in GenStat [50] to account for the split-plot blocking structure of the field trials.

The re-analysis of the field trial data included an additional factor identifying the presence/absence of *TaASN-B2*, with the effect of variety nested within the *TaASN-B2* factor. The trials in the individual years were analysed according to the designs indicated before [11] using ANOVA, with the addition of the *TaASN-B2* factor. The data combined across the two trials was analysed as a linear mixed model using the REML algorithm, allowing for the different design structures and sets of varieties included in the two trials, providing an overall comparison of both the presence/absence of *TaASN-B2*, and the differences between the varieties included across the two trials (allowing comparisons of varieties included in different years).

## Supplementary Information

**Additional file 1 **Fig. S1. Allelic diversity in TaASN1. Predicted amino acid sequence of the full-length ASN-B1 protein encoded by varieties Robigus, Julius, Norin 61, Mace and Spelt wheat (wild-type), compared to the truncated protein encoded by varieties CDC Landmark, Claire, Jagger, Cadenza, Paragon, Arina, CDC Stanley and Lancer (ASN-B1 truncation). In the latter varieties, a 16 bp deletion in exon 7 is predicted to introduce a premature stop codon at amino acid residue 375, indicated by *. The conserved GATase and ASN synthetase domains are highlighted. Fig. S2. PCR assay to distinguish presence and absence of *TaASN-B2* in a collection of 24 global wheat varieties. a. Schematic diagram of the assay to show primer positions and expected amplicon sizes. Amplification of a 189 bp product with primers P1 and P2 indicates that *TaASN B2* is deleted, while amplification of a 125 bp product with primers P3 and P4 indicates that *TaASN-B2* is present. One amplified fragment is expected in each reaction. b. Agarose gel electrophoresis of PCR products from the assay. Varieties with *TaASN-B2* deleted are highlighted in red, while varieties with the gene present are highlighted in green. A 100 bp ladder is shown in the first and last well of the gel for size comparison. Among the varieties are five carrying the *TaASN-B2* deletion and four with *TaASN-B2* present that were used to assay ASN expression in the grain. Full details of each variety are given in Table 2. Table S1. Natural variation in ASN proteins in wheat. For each protein, shades of green indicate that all amino acid substitutions are predicted to be tolerated and the encoded protein is predicted to be functional. Shades of yellow/orange indicate that at least one polymorphism is predicted to be disruptive for protein function based on SIFT analysis. Red indicates the gene is deleted in that variety. Full details of each protein type are provided in the key below the main table, where (T) indicates the amino acid substitution at that position is predicted to be tolerated, and (APF) indicates the change is predicted to affect protein function. Table S2. a. List of UK winter wheat (*Triticum aestivum*) varieties with *TaASN-B2* present or absent, separated by market class. b. List of common wheat varieties with *TaASN-B2* present or deleted among a panel of 24 global wheat varieties. ID, accession numbers and country of origin are provided. Table S3. Significance values for RT-qPCR and field analysis. a. ANOVA Analysis was performed using Timepoint*Variety*Homeologue as the treatment structure and Block/Subblock/Plot as the blocking structure. b. Significance values for factors in the ANOVA and REML analyses of field trial data. All analyses were performed on loge transformed data. ANOVA analyses were performed using Block/MainPlot/SplitPlot as the random model and (TaASN-B2/Variety)*Sulphur Treatment as the treatment model. REML analysis was performed using Year/Block/MainPlot/SplitPlot as the random model and Year*(TaASN-B2/Variety)*Sulphur Treatment as the treatment model. Table S4. Details of missing sequence data in ASN genes in some genome assemblies. Similarity among sequences was determined using all available sequence but some varieties had regions of ‘Ns’ within ASN genes, as indicated in the table below. Table S5. Primers used in this study.

**Additional file 2 **Mean TPM expression data for each *ASN* gene in RNA-seq datasets.

**Additional file 3.** Full, uncropped images of the gel electrophoresis results presented in this study.

## Abbreviations

ANOVA: Analysis of Variance
BLAST: Basic Local Alignment Search Tool
CRISPR: Clustered Regularly Interspersed Short Palindromic Repeats
CTAB: Cetyl Trimethyl Ammonium Bromide
DPA: Days Post Anthesis
EMS: Ethyl Methane Sulphonate
EU: European Union
FDA: Food and Drug Administration
FSANZ: Food Standards Agency of Australia and New Zealand
GA: Gibberellin
GATase: Glutamine amidotransferase
IWGSC: International Wheat Genome Sequencing Consortium
kb: kilo base pair
LTR: Long Terminal Repeat
PCR: Polymerase Chain Reaction
QTL: Quantitative Trait Locus
REML: Restricted Maximum Likelihood
TPM: Transcripts Per Million

## References

[1] Lea PJ, Sodek L, Parry MA, Shewry PR, Halford NG (2007). Asparagine in plants. Ann Appl Biol.

[2] Oddy J, Raffan S, Wilkinson MD, Elmore JS, Halford NG (2020). Stress, nutrients and genotype: understanding and managing asparagine accumulation in wheat grain. CABI Agric Biosci.

[3] Raffan S, Halford NG (2019). Acrylamide in food: progress in and prospects for genetic and agronomic solutions. Ann Appl Biol.

[4] IARC International Agency for Research on Cancer (1994). Some industrial chemicals; IARC monographs on the evaluation of carcinogenic risks to humans.

[5] European Commission (2017). Commission regulation EU. 2017/2158 establishing mitigation measures and benchmark levels for the reduction of the presence of acrylamide in food.

[6] Food and Drug Administration (2016). Guidance for Industry, Acrylamide in Foods.

[7] Zhivagui M, Ng AWT, Ardin M, Churchwell MI, Pandey M, Renard C, Villar S, Cahais V, Robitaille A, Bouaoun L, Heguy A, Guyton KZ, Stampfer MR, McKay J, Hollstein M, Olivier M, Rozen SG, Beland FA, Korenjak M, Zavadil J (2019). Experimental and pan-cancer genome analyses reveal widespread contribution of acrylamide exposure to carcinogenesis in humans. Genome Res.

[8] Raffan S, Oddy J, Halford NG (2020). The Sulphur response in wheat and its implications for acrylamide formation and food safety. Int J Mol Sci.

[9] Martinek P, Klem K, Vanova M, Bartackova V, Vecerkova L, Bucher P, Hajslova J (2009). Effects of nitrogen nutrition, fungicide treatment and wheat genotype on free asparagine and reducing sugars content as precursors of acrylamide formation in bread. Plant Soil Environ.

[10] Curtis TY, Powers SJ, Halford NG (2016). Effects of fungicide treatment on free amino acid concentration and acrylamide-forming potential in wheat. J Agric Food Chem.

[11] Curtis TY, Powers SJ, Wang R, Halford NG (2018). Effects of variety, year of cultivation and Sulphur supply on the accumulation of free asparagine in the grain of commercial wheat varieties. Food Chem.

[12] Rapp M, Schwadorf K, Leiser WL, Würschum T, Longin CFH (2018). Assessing the variation and genetic architecture of asparagine content in wheat: what can plant breeding contribute to a reduction in the acrylamide precursor?. Theor Appl Genet.

[13] Emebiri LC (2014). Genetic variation and possible SNP markers for breeding wheat with low-grain asparagine, the major precursor for acrylamide formation in heat-processed products. J Sci Food Agric.

[14] Raffan S, Halford NG (2021). Phylogenetic analysis of cereal asparagine synthetase genes. Ann Appl Biol.

[15] Xu H, Curtis TY, Powers SJ, Raffan S, Gao R, Huang J, Heiner M, Gilbert D, Halford NG (2018). Genomic, biochemical and modelling analyses of asparagine synthetases from wheat. Front Plant Sci.

[16] Dubcovsky J, Dvorak J (2007). Genome plasticity a key factor in the success of polyploid wheat under domestication. Science..

[17] Marcussen T, Sandve SR, Heier L, Spannag M, Pfeifer M, Jakobsen KS, Wulff BBH, Steuernage B, Mayer KFX, Olsen O-A, The International Wheat Genome Sequencing Consortium (2014). Ancient hybridizations among the ancestral genomes of bread wheat. Science.

[18] Gao R, Curtis TY, Powers SJ, Xu H, Huang J, Halford NG (2016). Food safety: structure and expression of the asparagine synthetase gene family of wheat. J Cereal Sci.

[19] Curtis TY, Raffan S, Wan Y, King R, Gonzalez-Uriarte A, Halford NG (2019). Contrasting gene expression patterns in grain of high and low asparagine wheat genotypes in response to Sulphur supply. BMC Genomics.

[20] Raffan S, Sparks C, Huttly A, Hyde L, Martignago D, Mead A, et al. Wheat with greatly reduced accumulation of free asparagine in the grain, produced by CRISPR/Cas9 editing of asparagine synthetase gene *TaASN2*. Plant Biotechnol J. 2021. 10.1111/pbi.13573. 10.1111/pbi.13573 PMC8384593 33638281

[21] Appels R, Eversole K, Feuillet C, Keller B, Rogers J, Stein N, The International Wheat Genome Sequencing Consortium (IWGSC) (2018). Shifting the limits in wheat research and breeding using a fully annotated reference genome. Science.

[22] Wicker T, Keller B (2007). Genome-wide comparative analysis of copia retrotransposons in Triticeae, rice, and *Arabidopsis* reveals conserved ancient evolutionary lineages and distinct dynamics of individual *copia* families. Genome Res.

[23] Wicker T, Matthews DE, Keller B (2002). TREP: A database for Triticeae repetitive elements. Trends Plant Sci.

[24] Choulet F, Alberti A, Theil S, Glover N, Barbe V, Daron J, Pingault L, Sourdille P, Couloux A, Paux E, Leroy P, Mangenot S, Guilhot N, Le Gouis J, Balfourier F, Alaux M, Jamilloux V, Poulain J, Durand C, Bellec A, Gaspin C, Safar J, Dolezel J, Rogers J, Vandepoele K, Aury JM, Mayer K, Berges H, Quesneville H, Wincker P, Feuillet C (2014). Structural and functional partitioning of bread wheat chromosome 3B. Science.

[25] Ramírez-González RH, Borrill P, Lang D, Harrington SA, Brinton J, Venturini L, Davey M, Jacobs J, van Ex F, Pasha A, Khedikar Y, Robinson SJ, Cory AT, Florio T, Concia L, Juery C, Schoonbeek H, Steuernagel B, Xiang D, Ridout CJ, Chalhoub B, Mayer KFX, Benhamed M, Latrasse D, Bendahmane A, Wulff BBH, Appels R, Tiwari V, Datla R, Choulet F, Pozniak CJ, Provart NJ, Sharpe AG, Paux E, Spannagl M, Bräutigam A, Uauy C, International Wheat Genome Sequencing Consortium (2018). The transcriptional landscape of polyploid wheat. Science.

[26] Hamilton NE, Ferry M (2018). ggtern: Ternary diagrams using ggplot2. J Stat Softw.

[27] Zadoks JC, Chang TT, Konzak CF (1974). A decimal code for the growth stages of cereals. Weed Res.

[28] Nirmal RC, Furtado A, Wrigley C, Henry RJ (2016). Influence of gene expression on hardness in wheat. PLoS One.

[29] Wicker T, Gundlach H, Spannagl M, Uauy C, Borrill P, Ramírez-González RH, De Oliveira R, Mayer K, Paux E, Choulet F, International Wheat Genome Sequencing Consortium (2018). Impact of transposable elements on genome structure and evolution in bread wheat. Genome Biol.

[30] Simsek S, Ohm JB, Lu H, Rugg M, Berzonsky W, Alamri MS, Mergoum M (2014). Effect of pre-harvest sprouting on physicochemical changes of proteins in wheat. J Sci Food Agric.

[31] Noree C, Sirinonthanawech N, Wilhelm JE (2019). *Saccharomyces cerevisiae ASN1* and *ASN2* are asparagine synthetase paralogs that have diverged in their ability to polymerize in response to nutrient stress. Sci Rep.

[32] Noree C, Monfort E, Shotelersuk V (2018). Human asparagine synthetase associates with the mitotic spindle. Biol Open.

[33] Middleton AM, Úbeda-Tomás S, Griffiths J, Holman T, Hedden P, Thomas SG, Phillips AL, Holdsworth MJ, Bennett MJ, King JR, Owen MR (2012). Mathematical modeling elucidates the role of transcriptional feedback in gibberellin signaling. Proc Natl Acad Sci U S A.

[34] Lei GJ, Fujii-Kashino M, Hisano H, Saisho D, Deng F, Yamaji N, Sato K, Zhao FJ, Ma JF (2020). Breeding for low cadmium barley by introgression of a Sukkula-like transposable element. Nat Food.

[35] European Food Safety Authority Panel on Contaminants in the Food Chain (CONTAM Panel) (2015). Scientific opinion on acrylamide in food. EFSA J.

[36] Krasileva KV, Vasquez-Gross HA, Howell T, Bailey P, Paraiso F, Clissold L, Simmonds J, Ramirez-Gonzalez RH, Wang X, Borrill P, Fosker C (2017). Uncovering hidden variation in polyploid wheat. Proc Natl Acad Sci U S A.

[37] Zhang Y, Pribil M, Palmgren M, Gao C (2020). A CRISPR way for accelerating improvement of food crops. Nat Food.

[38] Schmidt SM, Belisle M, Frommer WB (2020). The evolving landscape around genome editing in agriculture: many countries have exempted or move to exempt forms of genome editing from GMO regulation of crop plants. EMBO Rep.

[39] Bian X, Tyrrell S, Davey RP (2017). The grassroots life science data infrastructure.

[40] Blake VC, Woodhouse MR, Lazo GR, Odell SG, Wight CP, Tinker NA, et al. GrainGenes: centralized small grain resources and digital platform for geneticists and breeders. Database. 2019;baz065. 10.1093/database/baz065 PMC6580076 31210272

[41] Maccaferri M, Harris NS, Twardziok SO, Pasam RK, Gundlach H, Spannagl M, Ormanbekova D, Lux T, Prade VM, Milner SG, Himmelbach A, Mascher M, Bagnaresi P, Faccioli P, Cozzi P, Lauria M, Lazzari B, Stella A, Manconi A, Gnocchi M, Moscatelli M, Avni R, Deek J, Biyiklioglu S, Frascaroli E, Corneti S, Salvi S, Sonnante G, Desiderio F, Marè C, Crosatti C, Mica E, Özkan H, Kilian B, de Vita P, Marone D, Joukhadar R, Mazzucotelli E, Nigro D, Gadaleta A, Chao S, Faris JD, Melo ATO, Pumphrey M, Pecchioni N, Milanesi L, Wiebe K, Ens J, MacLachlan RP, Clarke JM, Sharpe AG, Koh CS, Liang KYH, Taylor GJ, Knox R, Budak H, Mastrangelo AM, Xu SS, Stein N, Hale I, Distelfeld A, Hayden MJ, Tuberosa R, Walkowiak S, Mayer KFX, Ceriotti A, Pozniak CJ, Cattivelli L (2019). Durum wheat genome highlights past domestication signatures and future improvement targets. Nat Genet.

[42] Solovyev V, Kosarev P, Seledsov I, Vorobyev D (2006). Automatic annotation of eukaryotic genes, pseudogenes and promoters. Genome Biol.

[43] Potter SC, Luciani A, Eddy SR, Park Y, Lopez R, Finn RD (2018). HMMER web server: 2018 update. Nucleic Acids Res.

[44] Sim NL, Kumar P, Hu J, Henikoff S, Schneider G, Ng PC (2012). SIFT web server: predicting effects of amino acid substitutions on proteins. Nucleic Acids Res.

[45] Murashige T, Skoog F (1962). A revised medium for rapid growth and bio assays with tobacco tissue cultures. Physiol Plant.

[46] Sambrook J, Fritsch EF, Maniatis T. Molecular cloning: a laboratory manual: CSHL Press; 1989.

[47] Chang S, Puryear J, Cairney J. A simple and efficient method for isolating RNA from pine trees. Plant Molec Biol Rep. 1993;11(2):113–6. 10.1007/BF02670468.

[48] Rieu I, Powers SJ (2009). Real-time quantitative RT-PCR: design, calculations, and statistics. Plant Cell.

[49] Ramakers C, Ruijter JM, Deprez RH, Moorman AF (2003). Assumption-free analysis of quantitative real-time polymerase chain reaction (PCR) data. Neurosci Lett.

[50] Genstat for Windows 21st Edition. VSN International, Hemel Hempstead, UK. 2020. https://Genstat.co.uk.

[51] R Core Team (2020). R: A language and environment for statistical computing.

[52] Kassambara A (2020). ggpubr: 'ggplot2' Based Publication Ready Plots. R package version 0.4.0.

[53] Xiang D, Quilichini TD, Liu Z, Gao P, Pan Y, Li Q, Nilsen KT, Venglat P, Esteban E, Pasha A, Wang Y, Wen R, Zhang Z, Hao Z, Wang E, Wei Y, Cuthbert R, Kochian LV, Sharpe A, Provart N, Weijers D, Gillmor CS, Pozniak C, Datla R (2019). The transcriptional landscape of polyploid wheats and their diploid ancestors during embryogenesis and grain development. Plant Cell.

[54] Pearce S, Vazquez-Gross H, Herin SY, Hane D, Wang Y, Gu YQ, Dubcovsky J (2015). WheatExp: an RNA-seq expression database for polyploid wheat. BMC Plant Biol.

[55] Warnes GR, Bolker B, Bonebakker L, Gentleman R, Huber W, Liaw A, Lumley T, Maechler M, Magnusson A, Moeller S, Schwartz M, Venables B (2013). gplots: Various R programming tools for plotting data 2013. R package version 2.12.1.

[56] Wickham H (2016). ggplot2: Elegant Graphics for Data Analysis.

