# Supplementary material for: Reduced free asparagine in wheat grain resulting from a natural deletion of TaASN-B2: investigating and exploiting diversity in the asparagine synthetase gene family to improve wheat quality
Source: BMC Plant Biol. 2021 Jun 29;21:302. doi: 10.1186/s12870-021-03058-7 (PMC8240372; doi:10.1186/s12870-021-03058-7)
Supplement: Supplementary file 1 — Additional file 1 Fig. S1. Allelic diversity in TaASN1. Predicted amino acid sequence of the full-length ASN-B1 protein encoded by varieties Robigus, Julius, Norin 61, Mace and Spelt wheat (wild-type), compared to the truncated protein encoded by varieties CDC Landmark, Claire, Jagger, Cadenza, Paragon, Arina, CDC Stanley and Lancer (ASN-B1 truncation). In the latter varieties, a 16 bp deletion in exon 7 is predicted to introduce a premature stop codon at amino acid residue 375, indicated by *. The conserved GATase and ASN synthetase domains are highlighted. Fig. S2. PCR assay to distinguish presence and absence of TaASN-B2 in a collection of 24 global wheat varieties. a. Schematic diagram of the assay to show primer positions and expected amplicon sizes. Amplification of a 189 bp product with primers P1 and P2 indicates that TaASN B2 is deleted, while amplification of a 125 bp product with primers P3 and P4 indicates that TaASN-B2 is present. One amplified fragment is expected in each reaction. b. Agarose gel electrophoresis of PCR products from the assay. Varieties with TaASN-B2 deleted are highlighted in red, while varieties with the gene present are highlighted in green. A 100 bp ladder is shown in the first and last well of the gel for size comparison. Among the varieties are five carrying the TaASN-B2 deletion and four with TaASN-B2 present that were used to assay ASN expression in the grain. Full details of each variety are given in Table 2. Table S1. Natural variation in ASN proteins in wheat. For each protein, shades of green indicate that all amino acid substitutions are predicted to be tolerated and the encoded protein is predicted to be functional. Shades of yellow/orange indicate that at least one polymorphism is predicted to be disruptive for protein function based on SIFT analysis. Red indicates the gene is deleted in that variety. Full details of each protein type are provided in the key below the main table, where (T) indicates the amino acid substi [file 12870_2021_3058_MOESM1_ESM.pdf]

Reduced free asparagine in wheat grain resulting from a natural deletion of *TaASN-B2*: investigating and exploiting diversity in the asparagine synthetase gene family to improve wheat quality

Joseph Oddy[1], Rocío Alarcón-Reverte[2], Mark Wilkinson[1], Karl Ravet[2], Sarah Raffan[1], Andrea Minter[3], Andrew Mead[3], J. Stephen Elmore[4], Isabel Moreira de Almeida[5], Nicholas C. Cryer[6], Nigel G. Halford[1], Stephen Pearce[2*]

1. Plant Sciences Department, Rothamsted Research, Harpenden, Hertfordshire, AL5 2JQ, United Kingdom

2. Department of Soil and Crop Sciences, Colorado State University, Fort Collins, CO 80523, USA

3. Computational and Analytical Sciences Department, Rothamsted Research, Harpenden, Hertfordshire, AL5 2JQ, United Kingdom

4. Department of Food & Nutritional Sciences, University of Reading, Whiteknights, Reading, RG6 6DZ, United Kingdom

5. Mondelez R&D International, Paris Saclay Tech Center, 6 Rue René Razel, 91400 Saclay, France

6. Mondelēz UK R&D Ltd, Bournville Lane, Bournville, Birmingham, B30 2LU, United Kingdom

[*] Corresponding author: Stephen Pearce, telephone 00 1 970 491 1427, email stephen.pearce@colostate.edu

Additional File 1 (.pdf)

Figures S1-S2, Tables S1-S5.

Fig. S1. Allelic diversity in *TaASN1*. Predicted amino acid sequence of the full-length ASN-B1 protein encoded by varieties Robigus, Julius, Norin 61, Mace and Spelt wheat (wild-type), compared to the truncated protein encoded by varieties CDC Landmark, Claire, Jagger, Cadenza, Paragon, Arina, CDC Stanley and Lancer (ASN-B1 truncation). In the latter varieties, a 16 bp deletion in exon 7 is predicted to introduce a premature stop codon at amino acid residue 375, indicated by *. The conserved GATase and ASN synthetase domains are highlighted.

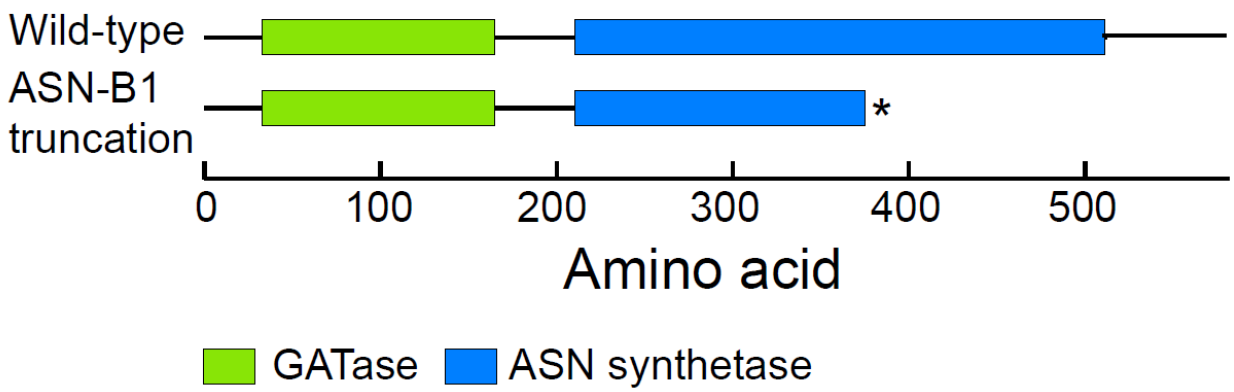

Fig. S2. PCR assay to distinguish presence and absence of *TaASN-B2* in a collection of 24 global wheat varieties. **a.** Schematic diagram of the assay to show primer positions and expected amplicon sizes. Amplification of a 189 bp product with primers P1 and P2 indicates that *TaASN-B2* is deleted, while amplification of a 125 bp product with primers P3 and P4 indicates that *TaASN-B2* is present. One amplified fragment is expected in each reaction. **b.** Agarose gel electrophoresis of PCR products from the assay. Varieties with *TaASN-B2* deleted are highlighted in red, while varieties with the gene present are highlighted in green. A 100 bp ladder is shown in the first and last well of the gel for size comparison. Among the varieties are five carrying the *TaASN-B2* deletion and four with *TaASN-B2* present that were used to assay *ASN* expression in the grain. Full details of each variety are given in Table 2.

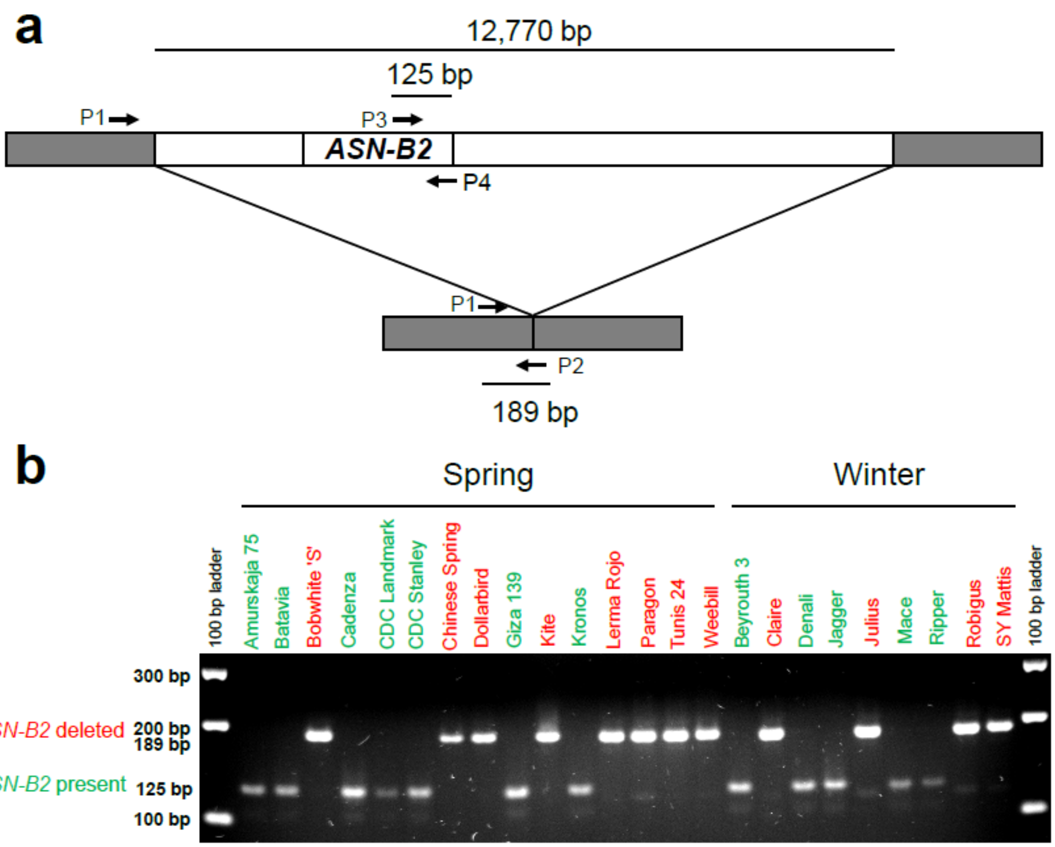

Table S1. Natural variation in ASN proteins in wheat. For each protein, shades of green indicate that all amino acid substitutions are predicted to be tolerated and the encoded protein is predicted to be functional. Shades of yellow/orange indicate that at least one polymorphism is predicted to be disruptive for protein function based on SIFT analysis. Red indicates the gene is deleted in that variety. Full details of each protein type are provided in the key below the main table, where (T) indicates the amino acid substitution at that position is predicted to be tolerated, and (APF) indicates the change is predicted to affect protein function.

Protein	IWGSC RefSeq v1.1 Gene ID	Robigus	CDC Landmark	Julius	Claire	Jagger	Cadenza	Paragon	Arina	Norin 61	CDC Stanley	SY Mattis	Lancer	Mace	Spelt
**TaASN-A1**	*TraesCS5A02G153900*														
**TaASN-B1**	*TraesCS5B02G152600*														
**TaASN-D1**	*TraesCS5D02G159100*														
**TaASN-A2**	*TraesCS3A02G077100*														
**TaASN-B2**	Not present														
**TaASN-D2**	*TraesCS3D02G077300*														
**TaASN-A3.1**	*TraesCS1A02G382800*														
**TaASN-B3.1**	*TraesCS1B02G408200*														
**TaASN-D3.1**	*TraesCS1D02G390500*														
**TaASN-A3.2**	*TraesCS1A02G422100*														
**TaASN-B3.2**	*TraesCS1B02G453600*														
**TaASN-D3.2**	*TraesCS1D02G430300*														
**TaASN-A4**	*TraesCS4A02G109900*														
**TaASN-B4**	*TraesCS4B02G194400*														
**TaASN-D4**	*TraesCS4D02G195100*														

Protein	RefSeqv1.0 gene ID*	Type 2	Type 3	Type 4
**TaASN-A1**	*TraesCS5A02G153900*	V19A (T)<br>R329S (APF)	R329S (APF)	
**TaASN-B1**	*TraesCS5B02G152600*	Y113F (T)<br>I529V (T)	STOP codon on 3rd exon	Not present
**TaASN-D1**	*TraesCS5D02G159100*	G268R (APF)		
**TaASN-A2**	*TraesCS3A02G077100*			
**TaASN-B2**	Not present	*		
**TaASN-D2**	*TraesCS3D02G077300*	R112S (T)<br>K131N (T)<br>P175L (T)<br>R418W (APF)		
**TaASN-A3.1**	*TraesCS1A02G382800*	G110R (APF)<br>S474P (T)<br>A559S (T)	R26_R31del<br>G110R (APF)<br>S474P (T)<br>A559S (T)	
**TaASN-B3.1**	*TraesCS1B02G408200*	K527E (T)	H565P (T)	
**TaASN-D3.1**	*TraesCS1D02G390500*			
**TaASN-A3.2**	*TraesCS1A02G422100*	A533T (T)<br>E537K (T)		
**TaASN-B3.2**	*TraesCS1B02G453600*	Not present		
**TaASN-D3.2**	*TraesCS1D02G430300*			
**TaASN-A4**	*TraesCS4A02G109900*	E439_P440Ins		
**TaASN-B4**	*TraesCS4B02G194400*			
**TaASN-D4**	*TraesCS4D02G195100*			

**ASN-B2* sequence based on Jagger reference genome.

Table S2. a. List of UK winter wheat (*Triticum aestivum*) varieties with *TaASN-B2* present or absent, separated by market class. **b.** List of common wheat varieties with *TaASN-B2* present or deleted among a panel of 24 global wheat varieties. ID, accession numbers and country of origin are provided.

a.

**UK winter wheat group**	***TaASN-B2* present**	***TaASN-B2* deleted**	
Group 1: Bread making	Avalon<br>Cadenza<br>Malacca	Crusoe<br>Gallant<br>Hereward<br>Shamrock	Skyfall<br>Solstice<br>Spark
Group 2: Bread making potential	Cashel<br>Einstein	Bonham<br>Charger<br>Chilton<br>Cordiale<br>Cubanita	Evoke<br>Podium<br>Rialto<br>Shango<br>Sterling
Group 3: Biscuit	Torch	Claire<br>Cocoon<br>Croft<br>Delphi<br>Diego<br>Icon<br>Invicta	Monterey<br>Robigus<br>Scout<br>Tuxedo<br>Warrior<br>Weaver<br>Zulu
Group 4: Soft	Lancaster	Alchemy<br>Cougar<br>Denman<br>Horatio<br>Panacea<br>Revelation	Leeds<br>Myriad<br>Rowan<br>Twister<br>Viscount
Group 4: Hard	Badger<br>Duxford<br>Kielder<br>Relay	Buster<br>Dickens<br>Evolution<br>Gator<br>Goldengun	Icebreaker<br>Oakley<br>Santiago<br>Savannah<br>Solace

b.

Growth habit	*TaASN-B2* present	Accession number	Country of origin	*TaASN-B2* deleted	Accession number	Country of origin
Spring	Amurskaja 75	PI 372145	Russia	Bobwhite 'S'		Mexico
	Batavia	PI-572700	Australia	Chinese Spring	CItr 14108	China
	Cadenza	id#39740	UK	Dollarbird	PI-525198	Australia
	CDC Landmark	id#39741	Canada	Kite	PI-386162	Australia
	CDC Stanley	id#39742	Canada	Lerma Rojo	CItr 13651	Mexico
	Giza 139	PI-185612	Egypt	Paragon	id#39749	UK
	Kronos	PI-576168	USA	Tunis 24	PI-278561	Tunisia
				Weebill	id#39754	Mexico
Winter	Beyrouth	PI 278533	Lebanon	Claire	id#39743	UK
	Denali	PI 664256	USA	Julius	id#39745	Germany
	Jagger	PI-593688	USA	Robigus	id#39751	UK
	Mace	id#39746	Australia	SY Mattis	id#39753	UK
	Ripper	PI 644222	USA			

Table S3. Significance values for RT-qPCR and field analysis. **a.** ANOVA Analysis was performed using Timepoint*Variety*Homeologue as the treatment structure and Block/Subblock/Plot as the blocking structure. **b.** Significance values for factors in the ANOVA and REML analyses of field trial data. All analyses were performed on $\log_e$ transformed data. ANOVA analyses were performed using Block/MainPlot/SplitPlot as the random model and (*TaASN-B2*/Variety)*Sulphur Treatment as the treatment model. REML analysis was performed using Year/Block/MainPlot/SplitPlot as the random model and Year*(*TaASN-B2*/Variety)*Sulphur Treatment as the treatment model.

a

**Factor**	**P-value**
Timepoint	<.001
Variety	<.001
Timepoint*Variety	<.001
Homeologue	<.001
Timepoint*Homeologue	<.001
Variety*Homeologue	<.001
Timepoint*Variety*Homeologue	<.001

b

	2011-2012 ANOVA	2012-2013 ANOVA	Combined REML
Treatment	0.037	0.010	<.001
TaASN-B2	<.001	0.236	0.027
*TaASN-B2**Variety	<.001	0.007	0.007
*TaASN-B2**Treatment	0.221	<.001	0.006
*TaASN-B2**Variety*Treatment	0.074	0.007	0.063
Year			<.001
Year**TaASN-B2*			0.951
Year*Treatment			<.001
Year**TaASN-B2**Treatment			0.004
Year**TaASN-B2**Variety			0.300
Year**TaASN-B2**Variety*Treatment			0.192

Table S4: Details of missing sequence data in *ASN* genes in some genome assemblies. Similarity among sequences was determined using all available sequence but some varieties had regions of 'Ns' within *ASN* genes, as indicated in the table below.

Gene	IWGSC RefSeq v1.1 Gene ID	Information on sequence availability
TaASN-A1	*TraesCS5A02G153900*	Full-length sequence in all varieties.
TaASN-B1	*TraesCS5B02G152600*	Full-length sequence in all varieties.
TaASN-D1	*TraesCS5D02G159100*	Full-length sequence in all varieties.
TaASN-A2	*TraesCS3A02G077100*	Full-length sequence in all varieties.
TaASN-B2	*Not present*	Full-length sequence in all varieties.
TaASN-D2	*TraesCS3D02G077300*	Cadenza - missing sequences in exons 10 and 11.
TaASN-A3.1	*TraesCS1A02G382800*	Full-length sequence in all varieties.
TaASN-B3.1	*TraesCS1B02G408200*	Full-length sequence in all varieties.
TaASN-D3.1	*TraesCS1D02G390500*	Full-length sequence in all varieties.
TaASN-A3.2	*TraesCS1A02G422100*	Robigus and Claire – missing sequence from exons 1-3.<br>Julius, Jagger, Arina, Lancer, Mace and Spelt – missing sequence from exon 1.<br>Cadenza - missing sequence at the end of exon 1.<br>Paragon - missing sequence in intron 3*.
TaASN-B3.2	*TraesCS1B02G453600*	Robigus, Cadenza and Paragon - missing sequence in exon 4.<br>Spelt - missing sequence in exon 1.
TaASN-D3.2	*TraesCS1D02G430300*	Robigus, Claire and Paragon - missing sequence in intron 3*
TaASN-A4	*TraesCS4A02G109900*	Paragon and Norin 61 - missing sequence in exon 1.
TaASN-B4	*TraesCS4B02G194400*	Full-length sequence in all varieties.
TaASN-D4	*TraesCS4D02G195100*	Full-length sequence in all varieties.

* Intron 3 contains some sequence similarity to exon 4, although in each variety, missing sequence means it is not possible to determine whether this is a complete copy.

Table S5. Primers used in this study.

Primer name	Sequence (5'-3')
ASN-B2-Deletion-F	CGTATAGACCCCGACTCATTGG
ASN-B2-Deletion-R	GCGAGTTAAGGCATGAGCTAAATATC
ASN-2-Universal-F	CGCTCTACAACGAGGACAAG
ASN-2-Universal-R	CCAATGTAGAGAGGCGTGAC
ASN-B2_CS_F3 (P1*)	AGCAAGCCTTCACCATCATT
ASN-B2_CS_R1 (P2*)	GATGTAGGCATGTCAACGAGA
ASN-B2_qF1 (P3*)	AACAAGCCTGGGGTGATGAG
ASN-B2_qR1 (P4*)	TTGTCTCAAAAAGAAAAAGAACTTG
ASN-A2-F	TCAACGGGGAGGTCTACAAC
ASN-A2-R	GCAATGAAGCTGTTATCTCGTG
ASN-B2-F	GTCAACGGGGAGATCTACAACC
ASN-B2-R	GCGATGAAGCTGTGATCTCTTG
ASN-D2-F	GTGAACGGGGAGATTTACAAC
ASN-D2-R	GCAATGAAGCTCTTATCTCGTG
GAPDH-F	ACTTCCAGGGTGACAACAGG
GAPDH-R	GTGCTGTATCCCCCACTCGTT
PROSM-F	CGAGATCGACCAAGAATGG
PROSM-R	TGAGTGTGGCCTCCCTCC
SDH-F	GCTGCCATCATATCCATTCC
SDH-R	AGCAATGTTACCCCTCATCG

*P1-P4 denotes the primer names used to illustrate the assay in Additional file 1, Fig. S2.
