# Supplementary material for: Reduced free asparagine in wheat grain resulting from a natural deletion of TaASN-B2: investigating and exploiting diversity in the asparagine synthetase gene family to improve wheat quality
Source: BMC Plant Biol. 2021 Jun 29;21:302. doi: 10.1186/s12870-021-03058-7 (PMC8240372; doi:10.1186/s12870-021-03058-7)

Additional File 3. Full, uncropped images of the gel electrophoresis results presented in this study.

Figure 3a (part 1)

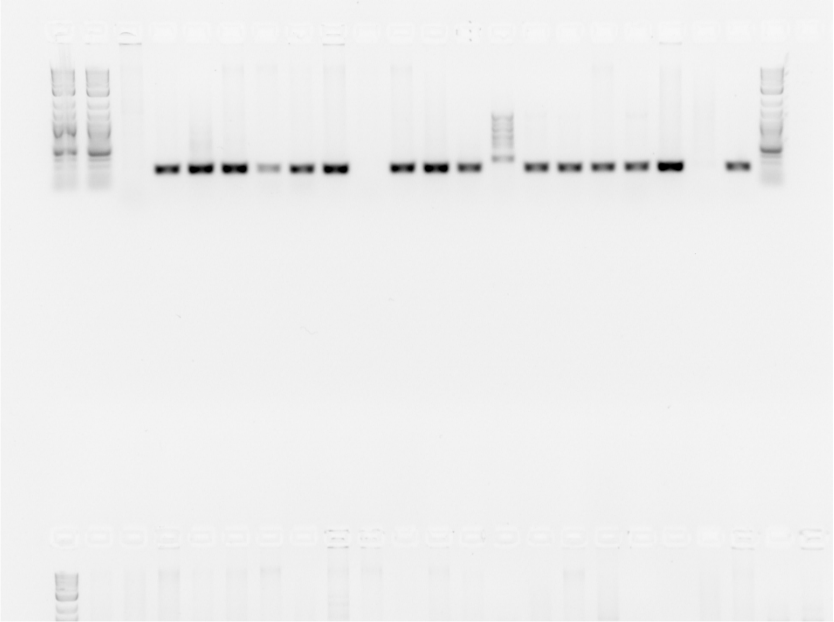

Figure 3a (part 2)

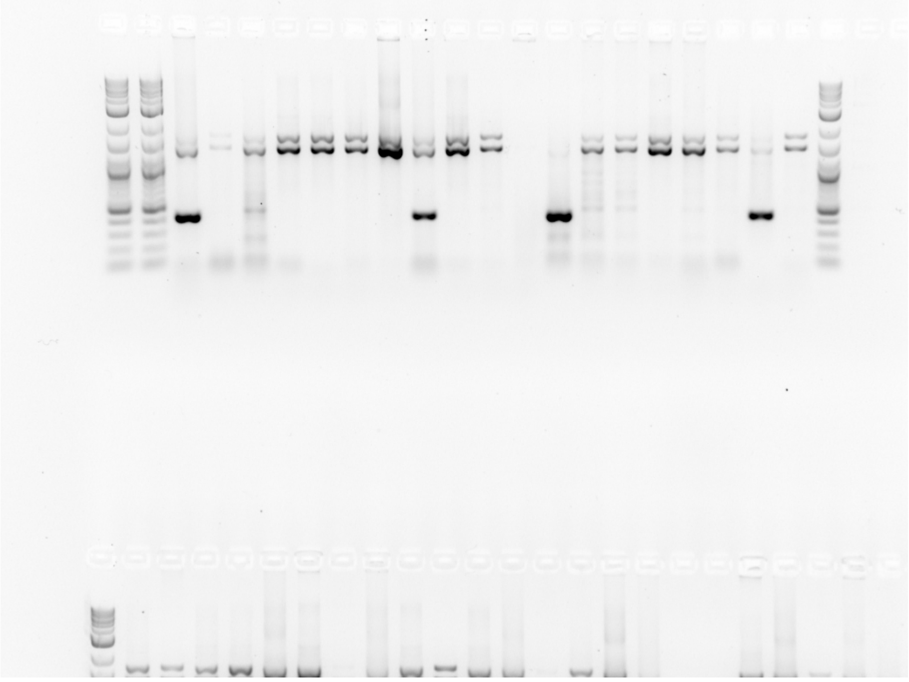

Figure 3b (part 1)

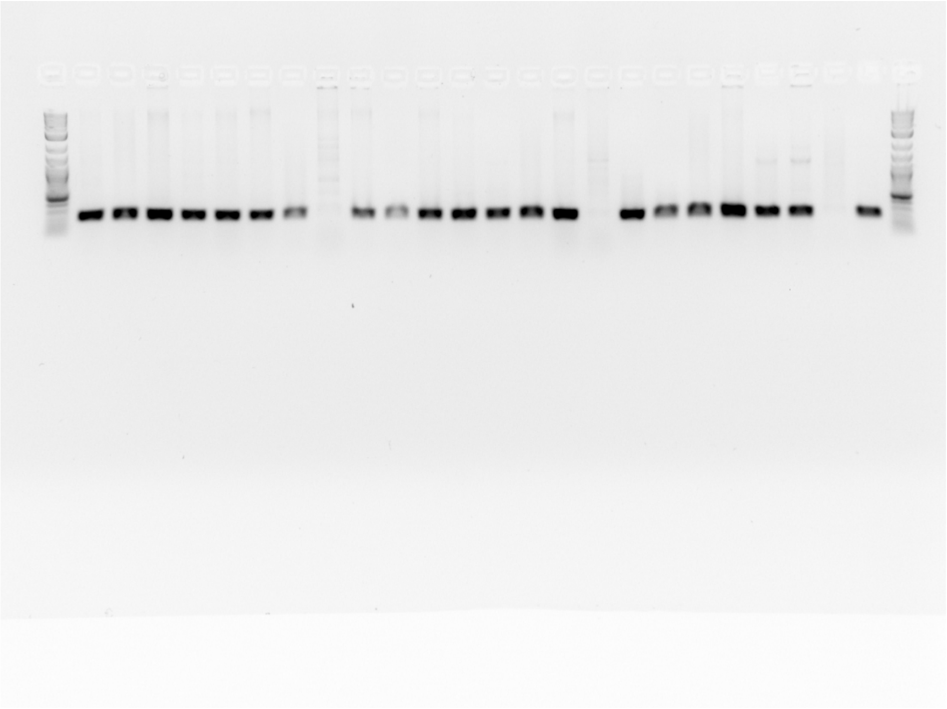

Figure 3b (part 2)

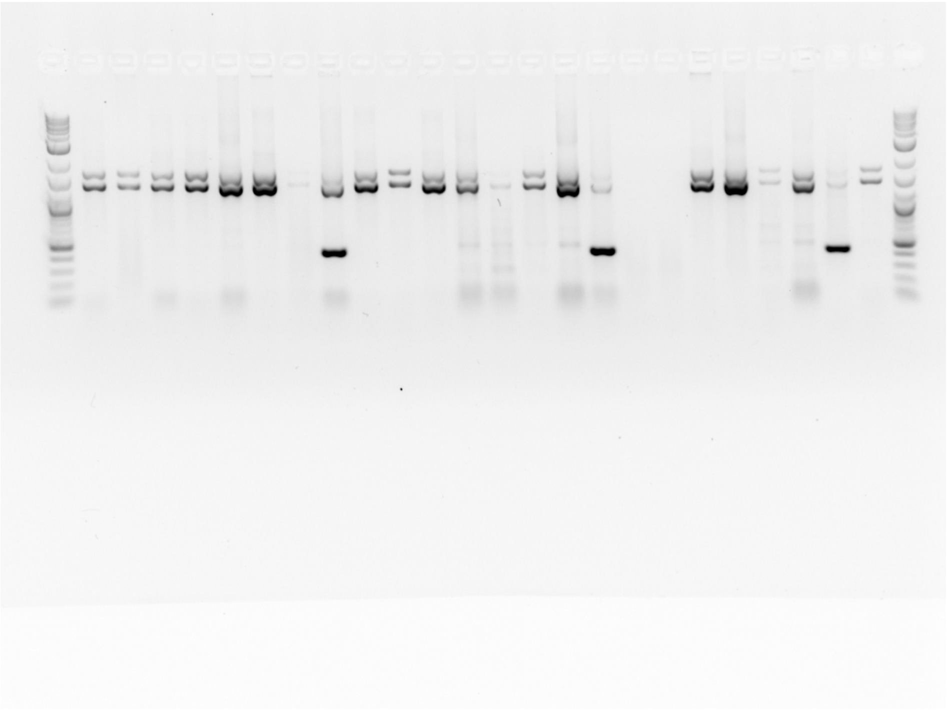

Figure 3c

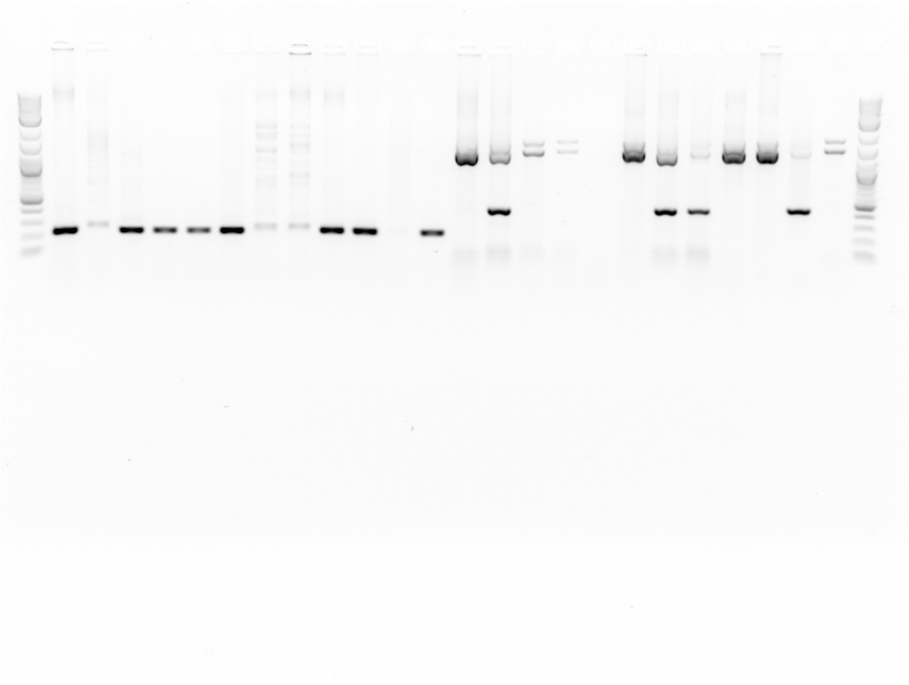

Figure 3d

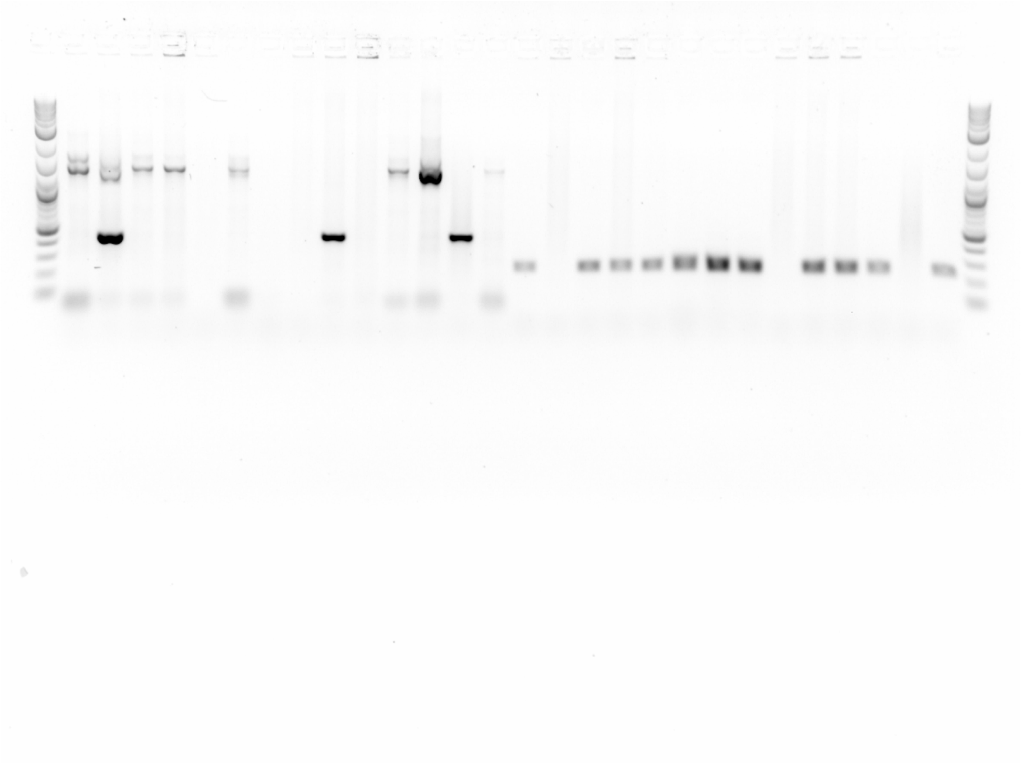

Figure 4

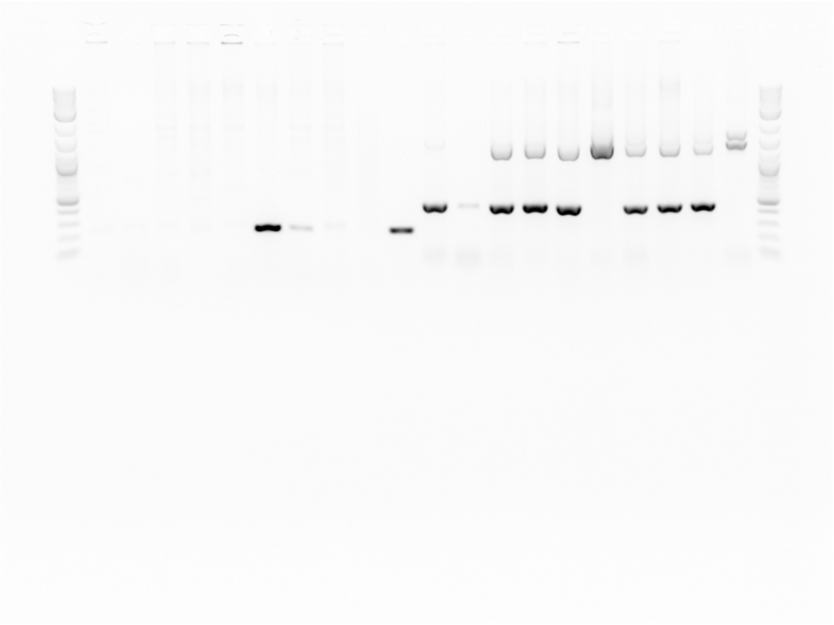

Additional file 1, Fig. S2b

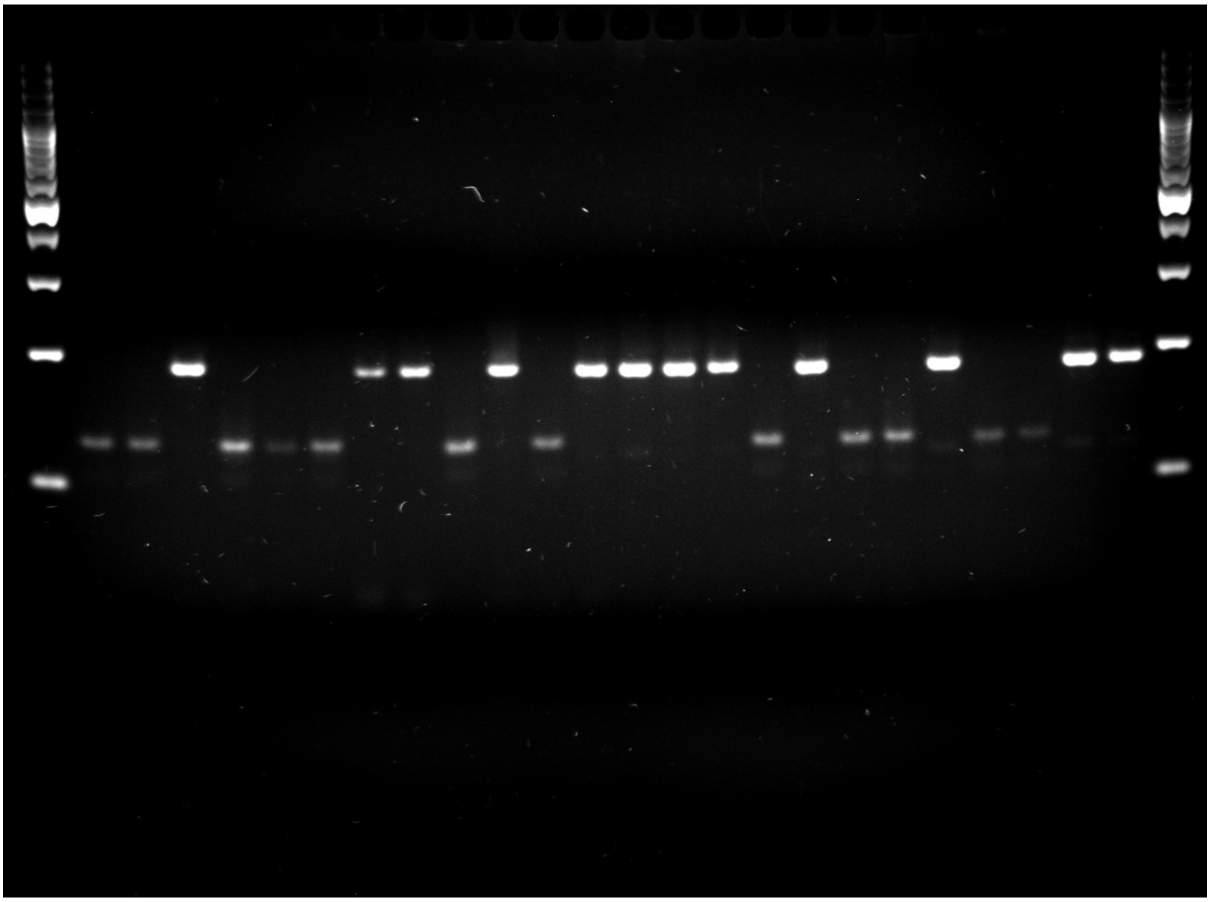

Supplement: Supplementary file 3 — Additional file 3. Full, uncropped images of the gel electrophoresis results presented in this study. [file 12870_2021_3058_MOESM3_ESM.pdf]
